# Synergistic Cancer Therapies Enhanced by Nanoparticles: Advancing Nanomedicine Through Multimodal Strategies

**DOI:** 10.3390/pharmaceutics17060682

**Published:** 2025-05-22

**Authors:** Seyed Mohamad Sadegh Mousavi-Kiasary, Ahmood Senabreh, Ashkan Zandi, Rogelio Pena, Frances Cruz, Ali Adibi, Nasrin Hooshmand

**Affiliations:** 1 Department of Physical and Environmental Sciences, Texas A&M University-Corpus Christi, Corpus Christi, TX 78412, USA; smousavikiasary@islander.tamucc.edu (S.M.S.M.-K.); asanabreh@islander.tamucc.edu (A.S.); rpena30@islander.tamucc.edu (R.P.); fcruz3@islander.tamucc.edu (F.C.); 2 School of Electrical and Computer Engineering, Georgia Institute of Technology, Atlanta, GA 30332, USA; ali.adibi@ece.gatech.edu; 3 Winship Cancer Institute, Emory University, Atlanta, GA 30322, USA

**Keywords:** nanoparticles, synergistic cancer therapy, targeted drug delivery, tumor microenvironment, multimodal treatment, personalized oncology, translational nanomedicine

## Abstract

Cancer remains a formidable global health challenge due to its complex pathophysiology and resistance to conventional treatments. In recent years, the convergence of nanotechnology and oncology has paved the way for innovative therapeutic platforms that address the limitations of traditional modalities. This review examines how nanoparticle (NP)-based strategies enhance the efficacy of chemotherapy, radiotherapy, phototherapy, immunotherapy, and gene therapy by enabling targeted delivery, controlled drug release, and tumor-specific accumulation via the enhanced permeability and retention (EPR) effect. We discuss the design and functionalization of various organic, inorganic, and hybrid NPs, highlighting their roles in improving pharmacokinetics, overcoming multidrug resistance, and modulating the tumor microenvironment. Particular emphasis is placed on dual and multimodal therapies, such as chemo-phototherapy, chemo-immunotherapy, and gene-radiotherapy, that leverage nanoparticle carriers to amplify synergistic effects, minimize systemic toxicity, and improve clinical outcomes. We also explore cutting-edge advances in gene editing and personalized nanomedicine, as well as emerging strategies to address biological barriers and immunosuppressive mechanisms in the tumor niche. Despite the undeniable promise of nanoparticle-based cancer therapies, challenges related to toxicity, scalable manufacturing, regulatory oversight, and long-term biocompatibility must be overcome before they can fully enter clinical practice. By synthesizing recent findings and identifying key opportunities for innovation, this review provides insight into how nanoscale platforms are propelling the next generation of precision oncology.

## 1. Introduction

Cancer remains one of the most complex and formidable challenges in modern medicine, with a steadily increasing incidence and mortality rate worldwide. In 2022 alone, an estimated 20 million new cancer cases and 9.7 million cancer-related deaths were reported globally [1]. According to projections by the American Cancer Society, the burden in the United States is expected to reach 2,001,140 new diagnoses and 611,720 cancer-related deaths in 2024 [2,3]. The fundamental complexity of cancer arises from its heterogeneous pathophysiology, which includes genetic mutations, cellular plasticity, and interactions with the tumor microenvironment (TME), making it difficult to treat with conventional methods alone [4].

Traditional cancer treatment modalities include surgery, chemotherapy, radiotherapy, hormone therapy, targeted therapy, phototherapy, and immunotherapy. Each of these approaches has contributed to significant advancements in cancer care, but continues to face critical limitations. Surgery remains the primary intervention for solid tumors; however, it is inherently invasive and often ineffective against metastatic disease. Surgical intervention may also inadvertently promote tumor cell dissemination by disrupting the TME, activating immunosuppressive pathways, and stimulating angiogenesis [5]. Additionally, the wound-healing process following surgery can create an environment that fosters residual tumor cell survival and metastasis through the extravasation of circulating tumor cells [6,7].

Chemotherapy is a widely utilized systemic treatment designed to target rapidly dividing cells. However, its lack of specificity results in off-target effects, damaging non-malignant proliferating cells and leading to significant side effects such as neurotoxicity, myelosuppression, and organ damage [8,9]. The emergence of multidrug resistance (MDR) remains a major obstacle, driven by mechanisms such as overexpression of ATP-binding cassette transporters and enhanced DNA repair pathways, which reduce drug efficacy [10,11]. Combination chemotherapy has been introduced to enhance therapeutic outcomes, but increased toxicity often limits its application [12,13].

Radiotherapy, another cornerstone of cancer treatment, utilizes ionizing radiation to induce DNA damage and inhibit tumor proliferation. However, due to its lack of specificity, it often results in off-target damage to surrounding healthy tissues, leading to acute and late toxicities, secondary malignancies, and genomic instability [14,15]. Although endocrine therapy provides a less toxic alternative for hormone receptor-positive cancers such as breast and prostate cancer, long-term use can result in osteoporosis and metabolic disorders [16,17].

Emerging treatment modalities, including immunotherapy, phototherapy, and gene therapy, are gaining momentum as innovative approaches to cancer care. Immunotherapy harnesses the body’s immune system through checkpoint inhibitors, monoclonal antibodies, and adoptive cell transfer, offering durable responses in some patients [18]. However, immune evasion by tumors, patient response variability, and immune-related adverse events present significant challenges [19]. Phototherapy, comprising photothermal therapy (PTT) and photodynamic therapy (PDT), provides a non-invasive cancer treatment that relies on light-activated agents for tumor ablation. Despite its specificity, the limited penetration depth of light restricts its application to superficial tumors [20,21]. Gene therapy aims to correct oncogenic mutations or silence aberrant gene expression, representing a promising avenue for personalized oncology. However, barriers such as unpredictable long-term effects, immune responses, and efficient gene delivery remain unresolved [22].

Given these limitations, there is a pressing need for strategies that enhance treatment efficacy while minimizing systemic toxicity. Nanotechnology has emerged as a transformative tool in oncology, providing novel solutions to improve cancer therapy. NPs, defined as materials ranging from 1 to 100 nm, exhibit unique physicochemical properties that make them highly advantageous for biomedical applications. These include a high surface area-to-volume ratio, tunable surface chemistry, and the ability to encapsulate and deliver therapeutic agents selectively [23,24]. The enhanced permeability and retention (EPR) effect allows nanoparticles to accumulate preferentially in tumor tissues due to leaky vasculature and impaired lymphatic drainage, providing a passive targeting mechanism that improves drug biodistribution and reduces off-target toxicity [25,26]. Also, surface modification of NPs with ligands such as antibodies, peptides, and aptamers enables active targeting, further improving specificity and minimizing systemic exposure [27,28].

Beyond drug delivery, nanoparticles facilitate synergistic interactions between therapeutic modalities. By acting as carriers for multiple agents, they enable combinatorial strategies such as chemo-phototherapy, chemo-immunotherapy, and chemo-radiotherapy, which enhance drug accumulation, increase radiosensitivity, and modulate immune responses for improved tumor eradication [29,30,31]. Furthermore, stimuli-responsive nanocarriers, which release drugs in response to pH, temperature, or enzyme activity, offer controlled spatiotemporal drug release, enhancing treatment precision [32]. Despite their immense potential, the clinical translation of nanomedicine faces several hurdles, including long-term biocompatibility, immune clearance, large-scale manufacturing, and regulatory challenges [33,34].

This review explores the role of nanoparticles in enhancing cancer therapy by providing an in-depth analysis of their classification, mechanisms of action, and capacity to improve treatment efficacy. Additionally, developing advancements in nanomedicine, challenges in clinical implementation, and future directions in personalized oncology are discussed. By synthesizing the latest developments in NP-based cancer treatment, this review aims to provide a comprehensive resource for researchers, clinicians, and industry professionals seeking to integrate nanotechnology into next-generation cancer therapies.

## 2. Nanoparticle Classification in Cancer Therapy

Enhancing treatment precision, drug delivery, and diagnostic capabilities, Nanoparticles have become integral to modern cancer therapy [35]. Broadly classified into organic, inorganic, and hybrid systems, each type offers distinct advantages. Organic nanoparticles, such as liposomes, polymeric nanoparticles, dendrimers, micelles, fluorescent organic nanoparticles, and protein-based nanoparticles, are widely adopted for their biocompatibility and ability to encapsulate various therapeutic agents. Liposomes and micelles improve the solubility of hydrophobic drugs and enable controlled release, while dendrimers and PLGA-based polymers provide high loading capacity and sustained drug delivery [36]. Fluorescent organic nanoparticles and protein-based systems, including albumin-bound carriers like Abraxane^®^, further support imaging and targeted delivery through receptor-mediated mechanisms [37,38,39]. Inorganic nanoparticles, including gold nanoparticles, magnetic nanoparticles, mesoporous silica, quantum dots, and carbon nanotubes, contribute unique structural and optical properties, enabling applications such as imaging, photothermal therapy, and magnetic targeting [40,41,42]. Gold nanoparticles, for example, are utilized in computed tomography and surface-enhanced Raman spectroscopy [43], while iron oxide particles aid magnetic resonance imaging [44]. Despite their utility, concerns about toxicity and long-term biocompatibility persist. Hybrid nanoparticles bridge the benefits of organic and inorganic systems [36]. Constructs like lipid-polymer hybrids and PEGylated metal-organic frameworks combine structural stability with biocompatibility to improve tumor targeting and immune evasion [45]. As nanomedicine advances, these multifunctional platforms are positioned to drive personalized, multimodal cancer treatment strategies with higher precision and therapeutic efficacy [46].

## 3. Multimodal Nanoparticle-Enhanced Combination Therapies

Advances in nanotechnology have enabled the development of multifunctional nanoparticle platforms that greatly amplify the therapeutic potency of existing cancer treatments. By leveraging size-dependent tumor accumulation, customizable surface chemistries, and controlled-release mechanisms, NPs can deliver multiple therapeutic agents in a targeted and coordinated manner. These capabilities open the door to synergistic treatment strategies where combining chemotherapy, phototherapy, radiotherapy, or immunotherapy leads to outcomes exceeding those of any single modality alone. In this section, we explore how NPs facilitate such combinatorial approaches, highlighting the ways they enhance drug delivery, overcome multidrug resistance, and modulate the tumor microenvironment for maximum therapeutic gain [47].

### 3.1. Chemotherapy, EPR-Driven Delivery, and Tumor Vasculature

Nanoparticles have been extensively explored for improving chemotherapy by enhancing drug delivery, increasing tumor penetration, and overcoming drug resistance. Traditional chemotherapy suffers from significant limitations, including poor bioavailability, systemic toxicity, and MDR, which limit therapeutic effectiveness [48]. The incorporation of nanoparticles into chemotherapy regimens aims to address these challenges by leveraging the EPR effect [49,50]. However, variations in EPR effect efficiency, driven by differences in endothelial permeability and tumor microenvironment heterogeneity, can influence treatment efficacy.

The EPR effect remains a foundational mechanism for nanoparticle-assisted chemotherapy, enabling passive tumor targeting by exploiting the abnormal vasculature of tumor tissues. Due to the presence of fenestrated endothelium and poor lymphatic drainage, tumors allow nanoparticles, typically 1 to 100 nm in size, to preferentially accumulate within the tumor interstitium [48]. This selective accumulation not only increases local drug concentration but also reduces systemic toxicity and improves therapeutic outcomes. Encapsulation of chemotherapeutic agents within nanoparticles further enhances drug solubility, stability, and circulation time, allowing more effective delivery to hypoxic or otherwise hard-to-reach tumor regions [49,50].

However, passive targeting via the EPR effect alone is not always sufficient, particularly in the context of small metastatic lesions or tumors with heterogeneous and poorly vascularized regions. In such cases, active targeting strategies offer a more precise alternative. By functionalizing nanoparticles with ligands such as antibodies, peptides, aptamers, hyaluronic acid, or folate, they can bind selectively to overexpressed receptors on cancer cells, such as HER2, CD44, or folate receptors, thereby promoting receptor-mediated endocytosis and improving intracellular drug delivery and therapeutic efficacy [51,52,53,54,55]. Moreover, targeting abnormal tumor vasculature or the compromised blood–brain barrier has shown potential in overcoming limitations associated with EPR alone, enabling more efficient drug penetration and restoration of tissue-specific physiological function. Recent studies have highlighted the role of active transcytosis pathways as an additional mechanism, suggesting that nanoparticle accumulation in tumors may not rely solely on passive diffusion [56].

**Figure 1:**
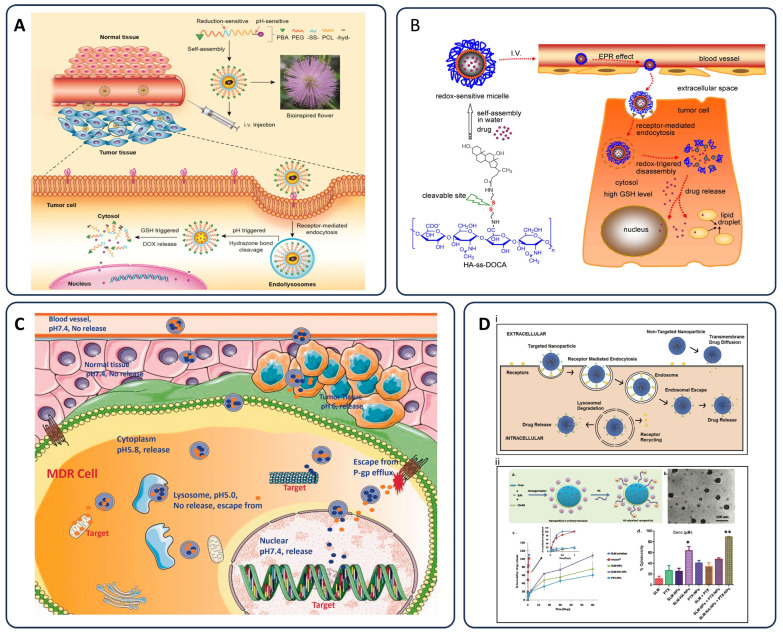
(**A**) Schematic depicting polymeric micelles self-assembled from PBA-PEG-SS-PCL-hyd-DOX conjugates. Following intravenous administration, micelles undergo receptor-mediated endocytosis into tumor cells, where intracellular drug release is selectively triggered by elevated glutathione (GSH) levels and acidic intracellular environments; “Reprinted with permission from [63]. Copyright 2025 American Chemical Society”. (**B**) Schematic illustration highlighting self-assembly of hyaluronic acid-based redox-sensitive micelles (HA-ss-DOCA), their enhanced tumor accumulation via the EPR effect, and subsequent intracellular drug delivery. Internalized micelles undergo receptor-mediated endocytosis, lysosomal escape, and GSH-triggered disassembly to achieve controlled intracellular drug release; “Reprinted from [64] with permission from Elsevier”. (**C**) Mechanistic illustration of nano drug co-delivery system overcoming multidrug resistance. The nanoparticles selectively release their cargo within tumor cells, bypassing efflux mechanisms mediated by P-gp, and facilitate targeted delivery to nuclear or cytoplasmic therapeutic sites; “Adapted from [65]“. (**D**) (**D_i_**) The diagram contrasts drug release and cellular uptake between targeted and non-targeted PEGylated polymeric nanoparticles. Targeted particles enter cells via receptor-mediated endocytosis. In contrast, non-targeted particles may release drugs into the extracellular space, allowing passive diffusion across the membrane. (**D_ii_**) Overview of Paclitaxel and Salinomycin delivery platforms; (**a**) A schematic of SLM-HA-NP shows how DMAB imparts a positive surface charge, which is partially neutralized by hyaluronic acid. (**b**) TEM images confirm the spherical morphology and nanoscale size. (**c**) In vitro release profiles indicate full drug release of both SLM and PTX by day 60. (**d**) Cytotoxicity evaluation (via MTT assay) after 48 h shows varying toxicity across formulations, including free drugs, encapsulated drugs, targeted systems, and dual-loaded nanoparticles; * *p* < 0.01 in comparison with SLM, ** *p* < 0.01 in comparison with SLM + PTX. “Adapted from [66]”.

To further improve tumor perfusion and nanoparticle uptake, both pharmacological and physical strategies are under investigation. Agents like nitric oxide, prostaglandins, bradykinin, peroxynitrite, and vascular endothelial growth factor (VEGF) can transiently increase vascular permeability, facilitating deeper nanoparticle penetration into tumors [57]. Similarly, physical approaches, such as localized ultrasound, radiation, hyperthermia, and photo-immunotherapy, temporarily disrupt endothelial junctions, increase blood flow, and promote extravasation of nanoparticles into tumor tissues [58,59]. These methods synergistically enhance nanoparticle-mediated chemotherapy by optimizing vascular access and improving drug distribution within the tumor microenvironment [60,61,62]. Recent innovations in stimuli-responsive nanoparticles have further advanced the precision of chemotherapy delivery. Wang et al. developed a highly tumor microenvironment-responsive micellar system based on PBA-PEG-SS-PCL-hyd-DOX conjugates, specifically engineered to respond to elevated intracellular glutathione *(GSH)* levels and acidic conditions. This platform self-assembles into micelles that remain stable in circulation but disassemble upon cellular internalization into tumor tissue, leading to site-specific doxorubicin release. Their work highlights the potential of redox-sensitive nanocarriers to enhance chemotherapeutic selectivity and mitigate systemic toxicity by leveraging the reductive tumor microenvironment (Figure 1A) [63]. Li et al. introduced HA-ss-DOCA micelles formed from amphiphilic hyaluronic acid-deoxycholic acid conjugates. These nanocarriers target CD44 receptors, commonly overexpressed in many tumors, while also exploiting redox-responsive disassembly. Following receptor-mediated endocytosis, intracellular *GSH* triggers the breakdown of disulfide linkages, enabling precise drug release. Figure 1B depicts the dual advantage of combining active targeting with intracellular redox sensitivity to improve the therapeutic index in tumor cells [64].

A major challenge in chemotherapy remains MDR, primarily driven by efflux proteins such as P-glycoprotein (P-gp), which expel drugs from cancer cells. Figure 1C illustrates how a nano drug co-delivery system can circumvent MDR by releasing its therapeutic payload directly into the cytoplasm or nucleus, where it exerts its action before being eliminated by P-gp [65]. Their system was designed to bypass the action of efflux pumps like P-glycoprotein (P-gp) by ensuring rapid release of both drugs directly into the cytoplasm or nucleus, where they can exert their pharmacological effects prior to being expelled. This design effectively prolongs drug retention inside resistant tumor cells and improves overall cytotoxicity, a promising direction for overcoming MDR in clinical settings.

Lastly, the success of nanoparticle-mediated drug delivery also hinges on the targeting strategy employed. Figure 1D compares targeted and non-targeted nanoparticles in terms of delivery efficiency. Targeted systems are equipped with ligands that bind specific cancer cell receptors, leading to receptor-mediated endocytosis and intracellular drug release. In contrast, non-targeted nanoparticles rely on passive diffusion following extracellular release, a less efficient route often accompanied by increased off-target toxicity and reduced therapeutic impact [66].

### 3.2. Photothermal and Photodynamic Therapies

Cancer treatment has traditionally relied on surgery, chemotherapy, and radiotherapy, yet these methods are often associated with severe side effects, off-target toxicity, and incomplete tumor eradication. To address these challenges, light-based therapies, such as PTT and PDT, have emerged as strong alternatives. These minimally invasive strategies use nanoparticles to enhance tumor targeting, improve therapeutic precision, and minimize damage to healthy tissues. Both PTT and PDT rely on external light activation to selectively destroy cancer cells, offering a degree of control that conventional therapies lack [67]. PTT utilizes photothermal agents to convert near-infrared (NIR) light into heat, effectively ablating cancerous tissues. In contrast, PDT employs photosensitizers that generate reactive oxygen species (ROS) upon light exposure, inducing oxidative stress-mediated cell death [68,69].

Despite their promise, both therapies face limitations, such as limited light penetration in deep-seated tumors and challenges in oxygen-dependent PDT. The integration of NPs has significantly improved the efficiency of these treatments, allowing for enhanced light absorption, improved stability of therapeutic agents, deeper tissue penetration, and more precise tumor targeting. Moreover, combining PTT and PDT with chemotherapy, a strategy known as chemo-phototherapy, further enhances therapeutic efficacy by leveraging multiple mechanisms of tumor destruction. This section explores how nanoparticle-mediated PTT and PDT enhance the selectivity and potency of light-based therapies, their underlying mechanisms, and how their synergistic combination with chemotherapy holds great potential for next-generation cancer treatment [70].

#### 3.2.1. Photothermal Therapy (PTT)

PTT utilizes nanoparticles that absorb light energy and convert it into heat, leading to localized tumor destruction. The key advantage of PTT lies in its ability to employ near-infrared (NIR) light, which penetrates deeper into tissues compared to visible light, allowing for precise tumor targeting with minimal damage to surrounding healthy tissue [71]. Among the various nanomaterials explored for PTT, phosphorene nanosheets and quantum dots (QDs) have demonstrated significant potential due to their broad absorption spectrum in the visible and near-infrared range, enhancing their photothermal efficiency [72].

PTT relies on highly efficient photothermal agents such as gold nanorods, gold nanoshells, and carbon nanotubes, which are systemically introduced into the body and selectively accumulate in tumor sites. These nanoparticles have strong NIR absorption properties, making them ideal for targeted heat generation. When exposed to NIR radiation, these particles trap and convert light energy into heat, raising the temperature of the tumor microenvironment to therapeutic levels [73]. Tumor cells exposed to temperatures between 40–46 °C [74,75,76] experience protein denaturation, membrane disruption, and mitochondrial dysfunction, ultimately leading to apoptotic or necrotic cell death. The combination of these effects contributes to the overall cytotoxicity of PTT, making it an encouraging approach for treating solid tumors resistant to conventional therapies.

A significant aspect of PTT is its ability to modify tumor vasculature. The localized thermal effects alter the disorganized and hyperpermeable nature of tumor blood vessels, disrupting their architectural integrity and further enhancing the EPR effect [77]. This vascular disruption increases the tumor’s susceptibility to therapeutic agents by facilitating the deeper penetration of chemotherapeutic drugs and nanoparticles. The synergy between thermal ablation and enhanced drug delivery amplifies the overall efficacy of cancer treatment, making PTT an effective adjunct to chemotherapy and other therapeutic modalities [78].

**Figure 2:**
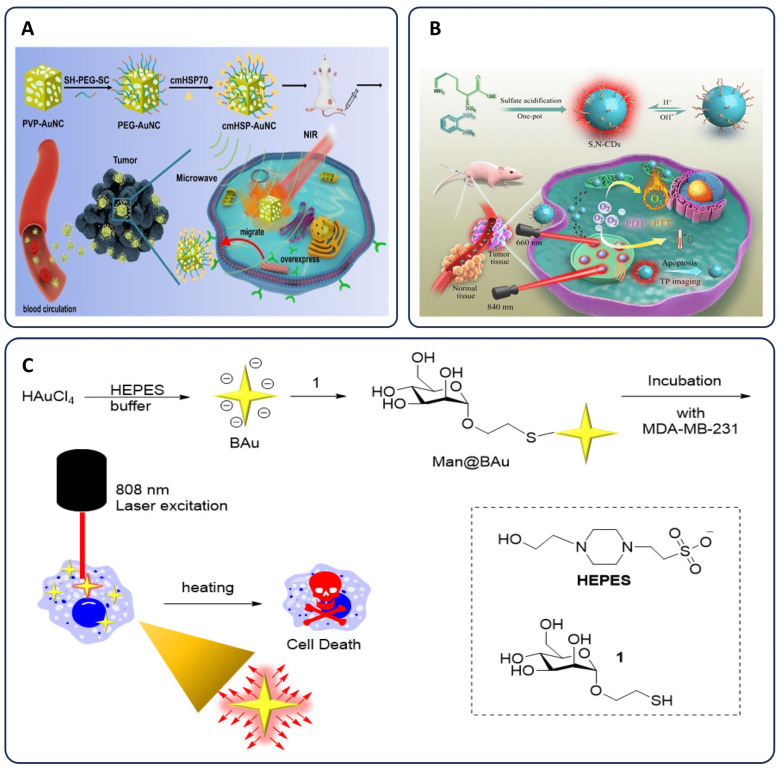
(**A**) Representation of cancer cell membrane-coated, heat shock protein-functionalized gold nanocages (cmHSP-AuNCs). These nanoparticles demonstrate tumor-targeted delivery and efficient photothermal therapy upon NIR irradiation; “Reprinted from [80], Copyright 2021, with permission from Elsevier” (**B**) Schematic illustrating the synthesis of sulfur/nitrogen co-doped carbon dots (S, N-CDs), their application for two-photon fluorescence imaging in response to pH, and synergistic photodynamic/photothermal therapy (PDT/PTT) for cancer treatment; “Reprinted (adapted) with permission from [86]. Copyright 2021 American Chemical Society”. (**C**) Illustration of mannose-functionalized gold nanoparticles (Man@BAu NPs) synthesized via covalent attachment of thiolated mannoside to gold nanoparticles formed in HEPES buffer. Upon exposure to near-infrared (NIR) laser irradiation (808 nm), the Man@BAu NPs exhibit photothermal conversion, elevating local temperature and inducing targeted cancer cell death; “Adapted from [81]”.

Advancements in nanotechnology have led to the creation of multifunctional nanoparticles tailored for targeted cancer therapy. One such development involves multilayered, surface amino-modified single-band up-conversion nanoparticles (sb-UCNPs) conjugated with antibodies specific to breast cancer biomarkers like progesterone receptor (PR), estrogen receptor (ER), and human epidermal growth factor receptor 2 (HER2). These sb-UCNPs facilitate multiplexed molecular mapping and precise spatial profiling of biomarkers within breast cancer tissue [79]. Another innovation includes cancer cell membrane-coated, heat shock protein-functionalized gold nanocages (cmHSP-AuNCs). These cmHSP-AuNCs demonstrate tumor-targeted delivery and effective photothermal therapy upon near-infrared (NIR) irradiation. Additionally, pH, enzyme, and NIR-responsive immune-adjuvant nanoparticles (R837@MSN-mannose-AuNPs-Glu/Lys; RMmAGL) have been engineered to integrate tumor-specific photothermal therapy with photothermally assisted immunotherapy, enhancing antitumor efficacy (Figure 2A) [80].

Mannose-functionalized gold nanoparticles (Man@BAu NPs), produced by covalently attaching thiolated mannoside to gold nanoparticles formed in HEPES buffer, exhibit photothermal conversion upon exposure to NIR laser irradiation (808 nm), raising local temperatures and inducing targeted cancer cell death (Figure 2C) [81]. S,N-co-doped carbon dots–gold nanoparticles (S,N-CDs) have been developed to exploit elevated mitochondrial calcium levels in tumor cells, inducing calcium-dependent photothermal therapy and mitochondrial calcification-mediated starvation therapy, effectively suppressing tumors [82].

#### 3.2.2. Photodynamic Therapy (PDT)

PDT is a light-activated treatment that employs photosensitizing drugs to generate ROS, which selectively destroy cancer cells. Unlike PTT, which induces cell death through localized heating, PDT relies on oxidative stress-induced damage to cellular components such as lipids, proteins, and DNA. The generation of singlet oxygen (a highly reactive ROS) leads to irreversible cytotoxic effects, ultimately causing apoptosis, necrosis, or autophagy within tumor cells [83]. Photosensitizers, typically administered intravenously, accumulate preferentially in tumor tissues due to the EPR effect. Once activated by exposure to a specific wavelength of light, photosensitizers undergo an energy transfer process that converts molecular oxygen into ROS, initiating oxidative stress that disrupts critical cellular functions [84]. The primary mechanism of PDT-induced cell death involves the destruction of essential biomolecules within tumor cells, leading to membrane rupture, DNA fragmentation, and mitochondrial dysfunction. This multi-faceted cytotoxicity distinguishes PDT from conventional chemotherapy, making it particularly effective against chemoresistant cancers. However, PDT’s dependence on oxygen availability limits its efficacy in hypoxic tumor environments, where oxygen concentration is insufficient to sustain ROS production.

A major challenge of PDT is the limited penetration depth of visible light, which restricts its effectiveness to superficial tumors or lesions. Traditional photosensitizers absorb light in the visible spectrum, limiting the reach of PDT within deep-seated tissues. To overcome this limitation, researchers are exploring the use of near-infrared (NIR) light, which can penetrate deeper into tissues and excite photosensitizers more efficiently while minimizing collateral damage to surrounding healthy cells [85]. The development of up-conversion nanoparticles (UCNPs) and metal-organic frameworks (MOFs) has further enhanced PDT selectivity, allowing for precise light activation and improved photosensitizer stability. These innovations enable PDT to be applied in a broader range of cancers, including those in anatomically challenging locations.

Sulfur/nitrogen co-doped carbon dots (S,N-CDs) have been synthesized for two-photon fluorescence imaging responsive to pH variations and for combined photodynamic and photothermal therapy (PDT/PTT) in cancer treatment (Figure 2B) [86].

#### 3.2.3. Chemo-Phototherapy

Chemo-phototherapy integrates chemotherapy with PTT and PDT to enhance drug delivery, improve therapeutic outcomes, and reduce systemic toxicity. This combination overcomes several limitations of traditional chemotherapy, including poor tumor selectivity, systemic side effects, and drug resistance. By utilizing nanoparticles as carriers, chemo-phototherapy ensures localized drug release, controlled activation, and synergistic mechanisms that enhance tumor destruction while preserving healthy tissues [87]. One of the key advantages of this approach is the ability of PTT to increase vascular permeability, thereby improving the extravasation and penetration of chemotherapeutic agents within tumors. The heat generated by PTT not only destroys cancer cells but also modifies tumor vasculature, enhancing the uptake of chemotherapeutic drugs. Besides, PDT plays a complementary role by generating ROS that damage tumor cell structures and increase their susceptibility to chemotherapy-induced apoptosis [88,89].

The EPR effect is further amplified through PTT-induced vascular disruption. This mechanism facilitates the selective deposition of therapeutic agents within the tumor microenvironment, improving overall drug bioavailability [90]. Moreover, chemotherapeutic agents can serve as PDT sensitizers, promoting photosensitizer accumulation and enhancing ROS production, which further amplifies tumor cell destruction [91].

Nanoparticles play an essential role in chemo-phototherapy by acting as carriers for both chemotherapeutics and photosensitizers, ensuring their targeted release at the tumor site. Gold nanorods and gold nanoshells, due to their surface plasmon resonance (SPR) absorption of AuNPs in the near-infrared (NIR) region (1350–650 nm) absorption and photothermal conversion properties [92], have been widely used to mediate PTT-induced tumor ablation while simultaneously delivering chemotherapy drugs [93]. Similarly, UCNPs have been developed to overcome PDT’s penetration limitations by converting low-energy NIR light into higher-energy visible light, thereby activating photosensitizers deep within tumor tissues [94]. Recent advances in nanotechnology have led to the development of single-agent multifunctional nanoparticles that integrate chemotherapy and phototherapy into a single delivery system. These nanoparticles enable coordinated drug release, real-time imaging, and dual therapy activation, significantly improving treatment precision and reducing off-target effects [95]. The ability to combine chemotherapy with phototherapy using engineered nanoparticles represents a positive frontier in cancer treatment, providing an effective and minimally invasive strategy for tackling drug-resistant and aggressive tumors [96].

##### Synergistic Mechanisms and Drug Uptake Enhancement

The combination of chemotherapy with PTT and PDT leverages synergistic mechanisms to improve drug uptake and maximize therapeutic efficacy. One of the key advantages of this approach is PTT-induced tumor permeability enhancement, which allows chemotherapeutic agents to penetrate deeper into the tumor microenvironment. The heat generated during PTT leads to vascular disruption, increasing the leakiness of tumor blood vessels and facilitating the extravasation of chemotherapeutic drugs into tumor tissues [88]. This effect is particularly beneficial for chemotherapeutic agents that suffer from poor bioavailability or limited tumor accumulation. Additionally, PDT contributes to drug uptake enhancement by ROS that compromise tumor cell integrity and weaken defense mechanisms, making cancer cells more susceptible to chemotherapy-induced apoptosis. This interaction between PDT and chemotherapy ensures a dual-attack mechanism, where ROS generation enhances DNA damage and disrupts cellular repair processes, further sensitizing tumor cells to chemotherapy [89].

Another major factor in improving drug delivery in chemo-phototherapy is the EPR effect. This phenomenon, which allows nanoparticles to accumulate preferentially in tumors due to their abnormal vasculature, is further amplified through PTT-induced vascular modulation. As a result, nanoparticles carrying both chemotherapeutic agents and photosensitizers achieve higher tumor-specific accumulation, leading to greater treatment efficacy with reduced systemic toxicity [90]. Beyond increasing drug uptake, PTT also contributes to microenvironment modulation, alleviating tumor hypoxia, a major factor limiting PDT effectiveness. The disruption of the disorganized vasculature allows for improved oxygenation, which is essential for effective ROS generation and enhanced PDT outcomes [91]. By integrating chemotherapy with nanoparticle-mediated PTT and PDT, chemo-phototherapy provides a multifaceted strategy to optimize drug penetration, maximize tumor destruction, and reduce adverse effects, thereby improving the overall therapeutic index.

##### Apoptosis Induction and Chemotherapy Sensitization

A critical advantage of chemo-phototherapy is its ability to induce apoptosis while sensitizing tumor cells to chemotherapy. PDT plays a pivotal role in this process by generating ROS upon light activation, leading to oxidative stress, mitochondrial dysfunction, and DNA damage within tumor cells. These effects weaken cellular repair mechanisms, making tumor cells more susceptible to chemotherapeutic agents [90].

The synergistic interaction between PDT and chemotherapy is particularly effective in amplifying apoptotic pathways. Chemotherapeutic agents, such as doxorubicin and cisplatin, cause DNA damage and disrupt cell cycle progression, while PDT-generated ROS further impair DNA repair proteins, ultimately triggering apoptosis. This dual-attack mechanism is beneficial in overcoming intrinsic resistance mechanisms that often limit the efficacy of chemotherapy alone [91]. Moreover, chemotherapy itself can act as a sensitizer for PDT, improving photosensitizer uptake and tumor response. Some chemotherapeutic agents enhance the intracellular accumulation of photosensitizers, ensuring their efficient activation upon light exposure. This pre-conditioning effect primes cancer cells for PDT-induced apoptosis, creating a feedback loop that maximizes therapeutic potency [93].

Nanoparticles further optimize this process by co-delivering chemotherapeutic agents and photosensitizers, ensuring synchronized release at the tumor site. By controlling drug loading, surface modifications, and release kinetics, nanoparticles facilitate a targeted and sustained therapeutic response, reducing off-target toxicity and enhancing tumor-selective apoptosis [94]. The integration of chemo-phototherapy with nanoparticle-mediated delivery systems represents a significant advancement in maximizing tumor cell apoptosis, overcoming drug resistance, and improving patient outcomes.

##### Nanoparticle Applications in Chemo-Phototherapy

Nanoparticles play a crucial role in chemo-phototherapy by enhancing drug delivery, optimizing treatment precision, and reducing systemic toxicity. Their ability to co-deliver chemotherapeutic agents and photosensitizers allows for a synchronized therapeutic response, ensuring that both modalities work synergistically to maximize tumor destruction [95]. Gold-based nanoparticles, particularly gold nanorods and nanoshells, are widely used due to their high photothermal conversion efficiency and near-infrared (NIR) light absorption properties [92,97,98,99,100]. These nanoparticles can generate localized heat for PTT while simultaneously serving as carriers for chemotherapeutic drugs, enabling a dual-function approach [101]. Gold nanoshells combined with liposomal doxorubicin have shown promising results in preclinical models, enhancing tumor regression while minimizing systemic side effects [100,102,103,104].

Another significant advancement is the use of UCNPs, which convert low-energy NIR light into higher-energy visible or ultraviolet light. This property allows for deep-tissue activation of PDT, overcoming the major limitation of conventional photosensitizers that require visible light for activation. Functionalized UCNPs ensure precise photosensitizer activation in deeply seated tumors, making PDT more effective for cancers located in hypoxic or poorly accessible regions [105,106]. Beyond traditional nanoparticles, theranostic nanoparticles are emerging as powerful tools in chemo-phototherapy. These multifunctional systems integrate diagnostic imaging capabilities with therapeutic functions, enabling real-time monitoring of drug distribution and treatment efficacy. By combining chemotherapy, PTT, and PDT within a single nanoparticle system, these advanced platforms offer unprecedented precision, reduced off-target effects, and improved patient outcomes [107]. The continuous evolution of nanoparticle-based strategies in chemo-phototherapy is paving the way for more effective, less invasive, and highly targeted cancer treatments. As research progresses, the integration of stimuli-responsive nanoparticles, hybrid nanocarriers, and personalized treatment approaches is expected to further enhance the potential of chemo-phototherapy in clinical applications [108].

##### Advanced Nanoparticle-Based Dual Therapy

Recent advancements in nanotechnology have led to the development of single-agent nanoparticles capable of integrating both chemotherapy and phototherapy within a single platform, offering a highly efficient dual-therapy approach. These engineered nanoparticles are designed to co-deliver chemotherapeutic agents and photosensitizers, ensuring a localized, synchronized treatment that maximizes tumor cell destruction while minimizing off-target effects [109].

One of the most reliable platforms includes gold nanorods and nanoshells, which act as both photothermal agents and drug carriers. When exposed to near-infrared (NIR) light, these nanoparticles generate localized heat for tumor ablation while simultaneously releasing chemotherapeutic drugs, enhancing their penetration and effectiveness [110]. This dual functionality reduces the required chemotherapy dosage, thereby minimizing systemic toxicity and improving patient tolerability. Another State-of-the-art approach is UCNPs, which overcome the penetration limitations of traditional photosensitizers by converting low-energy NIR light into visible or ultraviolet light. This property allows for deep-tissue PDT activation, making it a powerful tool for treating tumors located in difficult-to-reach areas. UCNPs can also be functionalized with ligands to improve tumor specificity and drug targeting, further enhancing therapeutic efficacy [111,112].

Hybrid nanocarriers, such as LPHNPs and MOFs, offer additional advantages by combining the biocompatibility of lipids with the stability and controlled release properties of polymers and inorganic materials. These hybrid systems enable precise spatiotemporal drug release, reducing systemic toxicity while maintaining a high therapeutic index [113]. By integrating chemotherapy and phototherapy into a single nanoparticle system, advanced dual therapy platforms provide a multifaceted approach to cancer treatment, improving drug bioavailability, tumor specificity, and overall treatment outcomes. As research progresses, these innovative nanoparticle-based systems are expected to revolutionize personalized cancer therapy, offering more precise and effective treatment options with reduced side effects [114].

**Figure 3:**
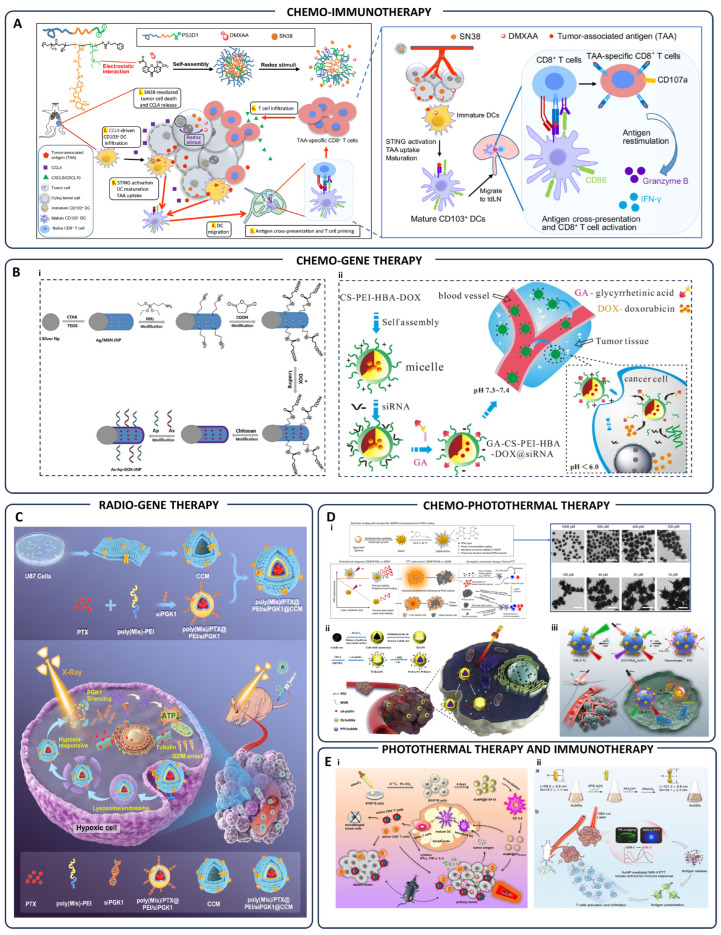
Nanoparticle-based combination cancer therapies integrating multiple conventional treatment modalities. (**A**) The mechanism and efficacy of PS3D1@DMXAA nanoparticles in breast and melanoma tumor models. Nanoparticles release SN38 and DMXAA within tumor cells via redox-responsive mechanisms, promoting tumor cell death, dendritic cell (DC) maturation, and cytotoxic CD8+ T-cell activation. This approach significantly suppresses primary tumors, reduces lung metastases, and enhances survival by modulating the tumor microenvironment and boosting antitumor immunity; “Reproduced from [115] with permission from, Copyright © 2020, The American Association for the Advancement of Science”. (**B**) (**B_i_**) Schematic of As-Ap-JNP synthesis via sol-gel methodology, surface functionalization with APTES and succinic anhydride yielding COOH-modified nanoparticles, and final conjugation with aptamer and antisense sequences via chitosan coating; “Reprinted from [116], Copyright 2025, with permission from Elsevier”. (**B_ii_**) Preparation of pH-responsive GA-CS-PEI-HBA-DOX@siRNA micelles enabling targeted and controlled co-delivery of doxorubicin (DOX) and siRNA to tumor sites for effective combination therapy; “Reprinted from [117], Copyright 2020, with permission from Elsevier”. (**C**) Illustration of the hypoxia-responsive RNAi nanomedicine mechanism, designed to silence PGK1 gene expression, thereby sensitizing orthotopic glioblastoma tumors to concurrent chemotherapy and radiotherapy; “Adopted from [118]”. (**D**) (**D_i_**) Design of PDA-coated spiky gold nanoparticles (SGNP@PDA) with TEM image, demonstrating enhanced photothermal stability and synergistic antitumor effects, effectively suppressing primary and distant tumors while establishing sustained immunological protection against tumor recurrence; “Adopted from [119]”. (**D_ii_**) Synthesis scheme and mechanism for Pt-HAuNS-PFH@O_2_ nanoparticles enhancing chemo-photothermal efficacy in breast cancer therapy; “Reprinted from [120], Copyright 2020, with permission from Elsevier”. (**D_iii_**) Representation of D/UCNP@cgAuNCs nanoassemblies engineered for tumor microenvironment-responsive ratiometric NIR-II fluorescence imaging and synergistic chemo-photodynamic therapy; “Adopted from [121]”. (**E**) (**E_i_**) Schematic preparation of AuNP@DCB16F10 nanoparticles, illustrating their mechanism of action for combined photothermal treatment and immune activation; “Adopted from [122]”. (**E_ii_**) (**a**) A schematic shows how gold nanorods (AuNRs) serve as seeds to grow gold nanodendrites (AuNDs), which exhibit a longitudinal LSPR peak in the NIR-II range. (**b**) Another illustration depicts the use of AuNDs for NIR-II photothermal therapy (PTT), enhancing the effectiveness of cancer immunotherapy; “Reprinted from [123], Copyright 2020, with permission from Elsevier”.

**Figure 4:**
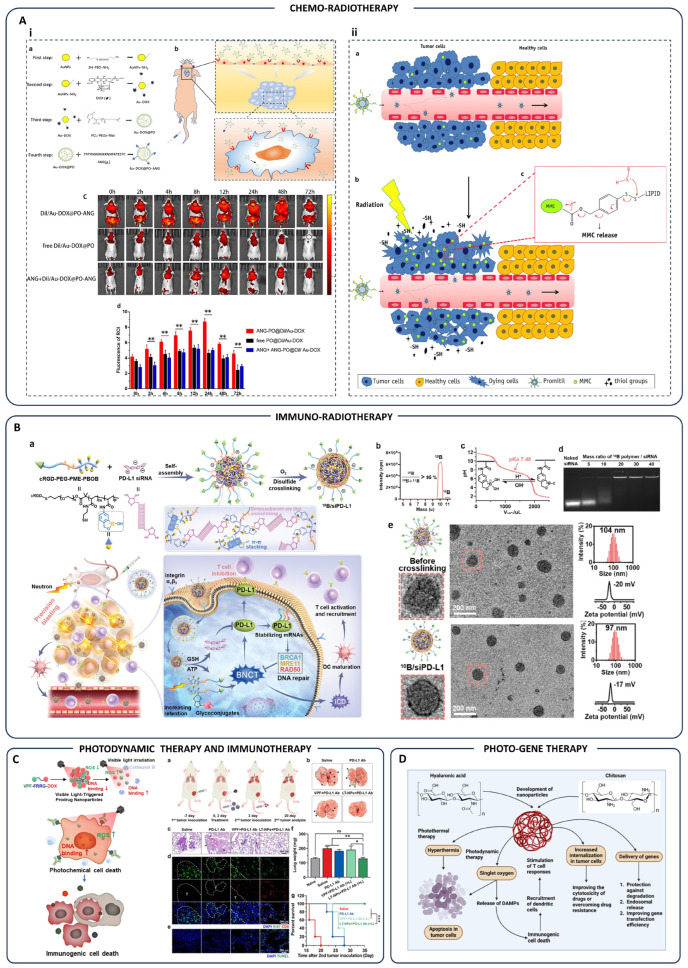
(**A**) (**A_i_**) (**a**) Diagram illustrating the synthesis process of Au-DOX@PO-ANG nanoparticles. (**b**) Schematic showing how Angiopep-2-conjugated, pH-sensitive polymersomes (Au-DOX@PO-ANG) are designed for targeted glioblastoma (GBM) therapy. (**c**) Biodistribution of Au-DOX@PO-ANG versus free Au-DOX@PO in tumor-bearing mice, with black circles marking tumor regions (ROIs). (**d**) Bar graph comparing fluorescence intensity in ROIs; ** *p* < 0.01 indicates statistical significance; “Adopted from [124]”. (**A_ii_**) Schematic overview illustrating how radiation influences mitomycin C (MMC) release from Promitil: (**a**) Promitil passively accumulates in tumors via the enhanced permeability and retention (EPR) effect. (**b**) Radiation triggers cancer cell death, releasing thiol-based reducing agents. (**c**) These agents cleave the dithiobenzyl linker in the lipid-based MMC prodrug, enabling controlled drug release at the tumor site; “Reprinted from [125], Copyright 2016, with permission from Elsevier”. (**B**) Design and evaluation of ^10^B/siPD-L1 nanoparticles: (**a**) Schematic shows the synthesis and use of ^10^B/siPD-L1 nanoparticles for combined boron neutron capture therapy (BNCT) and immunotherapy. (**b**) ICP-MS spectrum confirms boron isotope content in cRGD-PEG-PME-PBOB. (**c**) Acid–base titration reveals the buffering behavior of the copolymer. (**d**) Gel electrophoresis illustrates siRNA binding efficiency at varying ^10^B-to-siRNA mass ratios. (**e**) Particle size, morphology (via TEM), and surface charge (zeta potential) are compared before and after nanoparticle crosslinking; “Copyright 2025 Wiley. Used with permission from [126]”. (**C**) Metastasis suppression using LT-NPs (+L) combined with PD-L1 blockade in a bilateral CT26 tumor model: (**a**) The treatment schedule involved subcutaneous CT26 tumor implantation followed by intravenous injection to induce lung metastases. Mice received saline, anti-PD-L1, VPF (+L) with anti-PD-L1, or LT-NPs (+L) with anti-PD-L1 on days 0 and 2. Tumors were locally irradiated 6 h post-treatment. (**b**) Lung images were captured on day 20 to evaluate metastatic burden. (**c**–**e**) Lung sections were stained with H&E (**c**), Ki67 and CD8 (**d**), and TUNEL (**e**) to assess proliferation, immune infiltration, and apoptosis, respectively. (**f**) Lung weight was measured as a proxy for tumor load. (**g**) Survival curves showed treatment outcomes. Statistical significance (^ns^ *p* > 0.05, * *p* < 0.05, ** *p* < 0.01, *** *p* < 0.001) was analyzed using one-way ANOVA (**f**) and the log-rank test (**g**). “Adopted from [127]”. (**D**) Development of nanoparticles composed of chitosan and hyaluronic acid, facilitating synergistic photothermal and photodynamic therapies through hyperthermia induction and singlet oxygen generation. These nanoparticles stimulate dendritic cell recruitment, immunogenic cell death, T-cell activation, and improve intracellular delivery of therapeutic genes and drugs, augmenting overall anticancer efficacy; “Reprinted from [128], Copyright 2016, with permission from Elsevier.

Advanced nanoparticle-based dual therapies have recently emerged as highly effective cancer treatments by integrating multiple conventional therapeutic approaches into a single delivery platform. These multifunctional nanoparticles enable precise targeting and localized delivery of combined treatment modalities such as chemo-phototherapy, chemo-immunotherapy, radio-gene therapy, and photodynamic-immunotherapy, significantly amplifying therapeutic efficacy while minimizing off-target effects [3]. In chemo-immunotherapy, nanoparticles such as PS3D1@DMXAA simultaneously deliver chemotherapeutics and immune modulators, initiating tumor cell apoptosis and enhancing antigen presentation. This dual action results in robust dendritic cell activation and cytotoxic CD8+ T-cell responses, substantially suppressing tumor growth and metastasis [115] (Figure 3A). For chemo-gene therapy, innovative approaches like GA-CS-PEI-HBA-DOX@siRNA micelles and As-Ap-JNP nanoparticles combine chemotherapeutic agents with gene-silencing constructs. These nanoparticles enable targeted co-delivery of drugs and genetic material, overcoming multidrug resistance by effectively silencing resistance genes and enhancing chemotherapy sensitivity (Figure 3B) [116,117]. Radio-gene therapy utilizes nanoparticles engineered for hypoxia-triggered RNA interference, silencing critical tumor-promoting genes such as PGK1, thereby significantly sensitizing tumors like glioblastoma to concurrent chemotherapy and radiotherapy. This strategy improves tumor-specific radiosensitivity, greatly enhancing the therapeutic outcomes (Figure 3C) [118]. Chemo-photothermal therapy exploits nanoparticles like SGNP@PDA and Pt-HAuNS-PFH@O_2_, which synergistically combine photothermal agents and chemotherapy drugs. Upon near-infrared irradiation, these nanoparticles generate localized hyperthermia, augment chemotherapy drug penetration, and trigger sustained anti-tumor immune responses, effectively managing both primary and metastatic tumors (Figure 3D) [119,120,121]. Additionally, nanoparticles combining photothermal therapy with immunotherapy, such as AuNP@DCB16F10, potentiate immune activation by generating heat-induced immunogenic cell death and reshaping the immunosuppressive tumor microenvironment. This modality significantly enhances antitumor immune responses, providing effective control of both primary tumors and distant metastases (Figure 3E) [122,123]. In chemo-radiotherapy, nanoparticles such as Au-DOX@PO-ANG and Promitil nanoparticles enhance the selective accumulation and controlled release of chemotherapeutics within tumor tissues, simultaneously sensitizing tumors to radiation-induced damage. This synergistic approach amplifies DNA damage, significantly improving therapeutic efficacy while reducing systemic toxicity (Figure 4A) [124,125]. Moreover, immuno-radiotherapy strategies, exemplified by 10B/siPD-L1 nanoparticles, integrate boron neutron capture therapy (BNCT) with immune checkpoint blockade, enabling precise delivery of therapeutic isotopes and immune-modulating siRNA to tumors. This combined modality improves antitumor immunity and radiation-induced cytotoxicity (Figure 4B) [126]. In photodynamic-immunotherapy, visible-light-triggered nanoparticles (LT-NPs) specifically accumulate in tumor tissue, generating reactive oxygen species upon irradiation. These nanoparticles, combined with anti-PD-L1 therapy, effectively induce immunogenic cell death and significantly enhance systemic antitumor immunity, preventing metastasis and tumor recurrence (Figure 4C) [127]. Finally, photo-gene therapy employing nanoparticles composed of chitosan and hyaluronic acid integrates photothermal and photodynamic effects with gene delivery. This combination initiates robust immune activation and significantly improves intracellular drug and gene uptake, representing a promising advancement in personalized cancer therapy (Figure 4D) [128]. To facilitate a clearer comparison of various nanoparticle-enabled combination therapies, Table 1 systematically integrates and organizes representative studies discussed above. It categorizes the modalities based on their therapeutic strategy, such as chemo-immunotherapy, radio-gene therapy, photothermal-immunotherapy, immuno-radiotherapy, and photo-gene therapy, while detailing the nanoparticle formulations, mechanisms of synergistic action, and tumor models used. By presenting these parameters side by side, the table enables direct comparison across different approaches, revealing how nanoparticle design is tailored to each therapeutic context. Notably, a key insight from the table is the diversity of mechanisms leveraged, from redox-responsive drug release to immune checkpoint blockade and hypoxia-adapted gene silencing, highlighting the strategic versatility of nanocarriers in overcoming the limitations of monotherapy and enhancing therapeutic precision.

**Table 1:** Overview and comparative analysis of recent nanoparticle-based dual therapy strategies.

Type of Therapy	Nanoparticle System	Mechanism of Action	Tumor Model/Samples	Ref.
Chemo-Immunotherapy	PS3D1@DMXAA nanoparticles	Redox-responsive release of SN38/DMXAA; DC maturation; CD8^+^ T-cell activation	Breast & melanoma tumors (4T1, B16F10)	[115]
Chemo-Gene Therapy	As-Ap-JNP nanoparticles	Aptamer/antisense targeted gene therapy; specific tumor targeting via functionalized surface	Tumor cells	[116]
	GA-CS-PEI-HBA-DOX@siRNA micelles	pH-sensitive delivery of DOX and siRNA; targeted co-delivery and gene silencing	Tumor cells	[117]
Radio-Gene Therapy	Hypoxia-responsive iRNA nanomedicine	PGK1 silencing under hypoxia; sensitization of glioblastoma to chemo-radiotherapy	Glioblastoma tumor cells	[118]
Chemo-Photothermal Therapy	PDA-coated spiky gold nanoparticles (SGNP@PDA)	Enhanced photothermal stability; tumor ablation; immunological memory induction	Primary & metastatic tumors	[119]
	Pt-HAuNS-PFH@O_2_ nanoparticles	Chemo-photothermal synergistic therapy; controlled O_2_/drug release	Breast cancer cells	[120]
	D/UCNP@cgAuNCs nanoassemblies	Tumor-responsive NIR-II imaging; synergistic chemo-photodynamic therapy	Tumor cells	[121]
Photothermal-Immunotherapy	AuNP@DCB16F10 nanoparticles	Photothermal tumor ablation; enhanced antitumor immune responses	Melanoma tumor cells	[122]
	Gold nanotheranostics	NIR-II photothermal therapy; targeted immunotherapy enhancement	Tumor-bearing animal models	[123]
Chemo-Radiotherapy	Au-DOX@PO-ANG polymersomes	Targeted drug delivery to glioblastoma; radiation-triggered drug release	Glioblastoma tumor-bearing mice	[124]
	Promitil nanoparticles (MMC prodrug)	Radiation-induced thiol-mediated MMC activation and tumor-specific cytotoxicity	Tumor models	[125]
Immuno-Radiotherapy	10B/siPD-L1 nanoparticles	Combined boron neutron capture therapy and PD-L1-targeted immunotherapy	Tumor cells/tissues	[126]
Photodynamic-Immunotherapy	LT-NPs (VPF-FRRG-DOX conjugates)	Cathepsin B-sensitive ROS generation; immunogenic cell death; PD-L1 blockade	Tumor-bearing mice	[127]
Photo-Gene Therapy	Chitosan/hyaluronic acid nanoparticles	Hyperthermia & photodynamic therapy; gene delivery; immune activation	Tumor cells	[128]

### 3.3. Immunotherapy in Cancer

Immunotherapy represents a paradigm shift in cancer treatment, leveraging the body’s own immune system to recognize and eliminate cancer cells. Unlike conventional therapies such as chemotherapy and radiotherapy, which directly target cancer cells, immunotherapy aims to modulate immune responses to enhance tumor destruction while minimizing damage to healthy tissues. However, tumors have developed sophisticated immune evasion mechanisms, including the expression of immune checkpoint proteins and the creation of an immunosuppressive TME. These challenges have led to the development of advanced immunotherapeutic strategies, including checkpoint inhibitors, cancer vaccines, and chemo-immunotherapy, many of which are significantly enhanced by nanotechnology [114].

#### 3.3.1. Checkpoint Inhibitors and Overcoming Immunosuppression

Checkpoint inhibitors are a class of immunotherapeutic agents that counteract immune suppression mechanisms exploited by tumors. Cancer cells evade immune surveillance by expressing inhibitory molecules such as programmed death-ligand 1 (PD-L1), which interacts with the programmed cell death protein 1 (PD-1) receptor on T-cells, effectively shutting down their anti-tumor activity. Checkpoint inhibitors, such as anti-PD-1 (e.g., pembrolizumab, nivolumab) and anti-PD-L1 antibodies, block these interactions, restoring T-cell function and enabling them to attack cancer cells [129]. However, despite their success in treating certain cancers, checkpoint inhibitors face significant challenges, including low response rates in some patients, immune-related adverse effects, and an immunosuppressive TME [130,131]. NPs offer innovative solutions to enhance checkpoint blockade therapy. By modulating the TME, improving drug delivery, and targeting immunosuppressive components, NPs can significantly boost the efficacy of checkpoint inhibitors. For instance, liposomes, polymeric NPs, and carbon nanotubes can be engineered to co-deliver checkpoint inhibitors along with cytokines or immune-stimulating agents to reprogram the TME, shifting it from immunosuppressive to immunostimulatory [132]. Additionally, nanoparticles can be functionalized with targeting ligands to precisely deliver checkpoint inhibitors to tumor-infiltrating immune cells, reducing systemic toxicity and enhancing treatment efficacy [133]. Nanoparticles can alter the hypoxic and metabolic conditions of the TME, which often hinder immune responses. Some advanced nanoparticle formulations have been designed to neutralize immunosuppressive factors, such as TGF-β and myeloid-derived suppressor cells (MDSCs), further promoting an effective anti-tumor immune response. By integrating checkpoint inhibitors with nanoparticle-based delivery, these strategies increase the duration of immune activation, improve response rates, and minimize off-target effects, making nano-enabled immunotherapy a promising frontier in cancer treatment [134,135].

**Figure 5:**
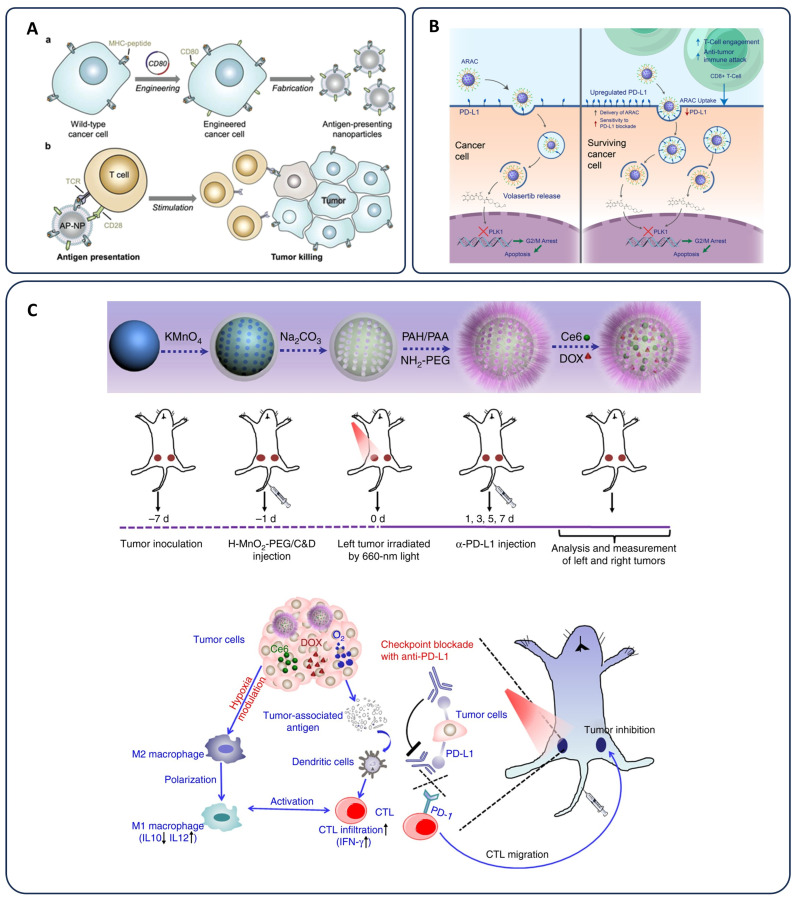
(**A**) Schematic illustrating the design of engineered cell-membrane-coated nanoparticles for direct antigen presentation: (**a**) Wild-type tumor cells, which naturally display antigens via MHC-I, are genetically modified to express CD80, a co-stimulatory molecule. Their membranes are then harvested and used to coat polymeric nanoparticle cores. (**b**) These antigen-presenting nanoparticles (AP-NPs) can directly activate tumor-specific T cells by engaging both the T cell receptor (TCR) and CD28. Once activated, the T cells target and eliminate cancer cells bearing the same antigens, thereby inhibiting tumor growth; “Adapted from [158]”. (**B**) Schematic of ARAC nanoparticles targeting PD-L1-expressing cancer cells. Initially, ARAC nanoparticles bind PD-L1, are internalized via receptor-mediated endocytosis, and release volasertib to induce G2/M arrest and apoptosis. Surviving cells upregulate PD-L1 expression, which, in turn, enhances subsequent ARAC nanoparticle targeting in a self-amplifying cycle. This ultimately leads to PD-L1 depletion, reactivating cytotoxic CD8+ T cells to mediate antitumor immunity; “Adapted from [136]”. (**C**) Schematic representation outlining the synthesis of hollow manganese dioxide nanoparticles (H-MnO_2_-PEG), dual drug loading, and combination therapy with anti-PD-L1 antibodies. Mechanistically, these nanoparticles remodel the tumor microenvironment, promoting polarization of macrophages towards an M1 phenotype and enhancing infiltration of cytotoxic T lymphocytes, thus potentiating anti-PD-L1-mediated immunotherapy; “Adapted from [137]”.

Antigen release agent and checkpoint inhibitor (ARAC) nanoparticles have been designed to target PD-L1-expressing cancer cells. Upon binding to PD-L1, these nanoparticles are internalized through receptor-mediated endocytosis, releasing volasertib to induce G2/M arrest and apoptosis. The surviving cancer cells upregulate PD-L1 expression, which enhances subsequent ARAC nanoparticle targeting in a self-reinforcing cycle. This process ultimately leads to PD-L1 depletion and reactivation of cytotoxic CD8+ T cells, thereby mediating antitumor immunity (Figure 5B) [136]. Hollow manganese dioxide nanoparticles (H-MnO_2_-PEG) have been synthesized for combination therapy with anti-PD-L1 antibodies. These nanoparticles remodel the tumor microenvironment by promoting the polarization of macrophages towards an M1 phenotype and enhancing the infiltration of cytotoxic T lymphocytes, thereby strengthening anti-PD-L1-mediated immunotherapy [137].

#### 3.3.2. Nanoparticle-Based Cancer Vaccines

Cancer vaccines aim to stimulate the immune system to recognize and destroy tumor cells by introducing tumor-associated antigens (TAAs) along with immune adjuvants. However, conventional cancer vaccines often suffer from poor antigen stability, inefficient uptake by antigen-presenting cells (APCs), and weak immune responses [133]. Nanoparticle-based delivery systems offer a solution by enhancing antigen presentation, protecting vaccine components from degradation, and enabling targeted delivery to immune cells [138]. Nanoparticles such as liposomes, virosomes, micelles, dendrimers, and nanogels have been developed as carriers for cancer vaccines, improving their immunogenicity and prolonging immune activation [139]. These nanosystems enable controlled release of antigens, ensuring sustained exposure to the immune system for an extended period [140]. One of the key advantages of nanovaccines is their ability to stimulate APCs, such as DCs, which are crucial for initiating a robust adaptive immune response [141].

A targeted approach in nanovaccine design involves functionalizing nanoparticles with specific ligands or antibodies to ensure selective delivery to dendritic cells. Once internalized, these nanoparticles facilitate efficient antigen processing and presentation via major histocompatibility complex (MHC) molecules, triggering the activation of cytotoxic T lymphocytes (CTLs) to eliminate tumor cells [142]. Besides, co-delivery of adjuvants such as toll-like receptor (TLR) agonists within nanoparticles enhances immune stimulation, boosting both innate and adaptive responses [143]. Personalized cancer vaccines have also gained traction, leveraging nanoparticles to carry patient-specific tumor antigens. This individualized approach improves vaccine efficacy by ensuring the immune system is trained to target unique tumor markers present in each patient [144]. By integrating nanotechnology with cancer immunotherapy, nanoparticle-based vaccines hold immense potential to overcome traditional vaccine limitations, enhance immune responses, and provide long-term protection against tumor recurrence. Another strategy employs engineered cell membrane-coated nanoparticles, termed antigen-presenting nanoparticles (AP-NPs). In this method, wild-type cancer cells expressing major histocompatibility complex class I (MHC-I) are genetically modified to express the co-stimulatory molecule CD80. Membranes derived from these modified cells are used to coat polymeric nanoparticles, enabling direct antigen presentation and simultaneous engagement of the T cell receptor (TCR) and CD28. This dual engagement activates T cells to target and eliminate cancer cells sharing the same antigens [145].

#### 3.3.3. Chemo-Immunotherapy and Immune Modulation

The combination of chemotherapy and immunotherapy (chemo-immunotherapy) is emerging as a powerful strategy in cancer treatment. While chemotherapy primarily works by directly killing tumor cells or inhibiting their proliferation, it can also modulate the TME to enhance immune response [146]. By inducing immunogenic cell death (ICD), chemotherapy promotes the release of TAAs, damage-associated molecular patterns (DAMPs), and pro-inflammatory cytokines, all of which enhance immune recognition and activation [147]. One of the key mechanisms by which chemotherapy enhances immunotherapy is through inducing ICD, which triggers an immune response by exposing tumor-specific antigens. Chemotherapeutic agents such as doxorubicin and oxaliplatin have been shown to stimulate ICD by promoting ATP secretion, high-mobility group box 1 (HMGB1) release, and calreticulin exposure, all of which enhance dendritic cell activation and subsequent T-cell priming [148]. This immune-stimulating effect improves the efficacy of immune checkpoint inhibitors, such as PD-1/PD-L1 and CTLA-4 blockers, by releasing T-cells from immune suppression [149].

Nanoparticles play a crucial role in enhancing chemo-immunotherapy by enabling targeted delivery of both chemotherapeutic agents and immunomodulatory molecules. Liposomes, polymeric nanoparticles, and lipid-based nanocarriers can encapsulate chemotherapeutic drugs along with immune-stimulating agents, ensuring precise co-delivery to the tumor site [150]. This approach reduces systemic toxicity while maximizing drug accumulation within the tumor, leading to enhanced immune activation. Functionalized nanoparticles carrying checkpoint inhibitors, cytokines, or siRNA targeting immunosuppressive genes can further modulate the immune landscape of tumors, overcoming resistance mechanisms and improving treatment outcomes [151]. By combining chemotherapy’s cytotoxic and immune-stimulating effects with immunotherapy’s ability to sustain long-term immune surveillance, chemo-immunotherapy represents a strong strategy to enhance antitumor responses, reduce tumor recurrence, and improve patient outcomes. With ongoing advancements in nanoparticle-based drug delivery systems, this multimodal approach continues to pave the way for more effective, personalized, and less toxic cancer treatments [152].

#### 3.3.4. Advances in Nanoparticle-Based Immunotherapy

Nanoparticle-based immunotherapy has improved cancer treatment by enhancing immune response, improving antigen presentation, and overcoming tumor immune evasion mechanisms. Engineered nanoparticles can be tailored to deliver immunotherapeutic agents precisely to the tumor site, reducing systemic toxicity and optimizing immune activation [153]. One of the major advancements in this field is the development of nanoparticle-based delivery systems for immune checkpoint inhibitors (ICIs) such as anti-PD-1/PD-L1 and anti-CTLA-4 antibodies. Traditional ICIs suffer from rapid clearance and off-target effects, but nanoparticle formulations help prolong their circulation time, enhance tumor accumulation, and improve therapeutic efficacy [154]. Nanoparticles functionalized with ligands or antibodies targeting tumor-associated immune cells can selectively modulate the TME by depleting immunosuppressive cells, such as regulatory T cells (Tregs) and MDSCs, thereby boosting anti-tumor immunity [155].

Another promising approach involves nanoparticle-mediated cytokine delivery. Cytokines like IL-2, IFN-γ, and GM-CSF play a pivotal role in immune activation but often cause severe side effects when administered systemically. Nanoparticles allow for controlled release and localized cytokine delivery, ensuring effective immune stimulation while minimizing toxicity [156]. CAR-T cell therapy, an innovative immunotherapy, has also benefited from nanotechnology. Nanoparticles can be used to enhance CAR-T cell expansion, improve targeting efficiency, and prolong persistence in the TME. Nanoparticle carriers can deliver gene-editing tools such as CRISPR/Cas9 to modify T cells for enhanced tumor recognition and cytotoxicity [157].

Recent innovations in personalized cancer vaccines have leveraged nanoparticle platforms to deliver tumor antigens and adjuvants, enhancing immune responses. These nano-vaccines are designed to stimulate DCs, which play a crucial role in T-cell activation. By optimizing antigen presentation, these nanoparticles can induce a strong and durable anti-tumor immune response, making them a promising strategy for personalized cancer immunotherapy. Biomimetic mini DC nanoparticles, shown in Figure 5A, are made by coating IL-2-loaded nanoparticles with membranes from tumor-primed dendritic cells. These “mini DCs” help activate T cells and improve the immune system’s ability to attack ovarian cancer tumors [158]. As research advances, nanoparticle-based immunotherapy continues to bridge the gap between immune activation and precision medicine, offering new opportunities for highly effective, patient-specific, and minimally toxic cancer treatments. The integration of nanotechnology into immunotherapy is expected to further refine cancer treatment strategies, enhance therapeutic outcomes, and expand the scope of immuno-oncology in clinical practice [159]. Improvement in cancer immunotherapy has happened by improving immune responses, facilitating antigen presentation, and addressing tumor immune evasion mechanisms. Engineered nanoparticles can be tailored to deliver immunotherapeutic agents precisely to tumor sites, thereby reducing systemic toxicity and optimizing immune activation. For instance, nanoparticle-based delivery systems have been developed for ICIs like anti-PD-1/PD-L1 and anti-CTLA-4 antibodies. Traditional ICIs often face challenges such as rapid clearance and off-target effects; however, nanoparticle formulations can prolong their circulation time, enhance tumor accumulation, and improve therapeutic efficacy (Figure 5C) [137]. Also, the physicochemical properties of PLGA nanoparticles (PLGA-NPs) have been characterized. Synthesis strategies for R848-loaded PLGA-NPs and bispecific antibody-functionalized bis-R848-PLGA-NPs have been evaluated. Analyses of hydrodynamic diameter, polydispersity index (PDI), and zeta potential for various formulations have been conducted. Morphological characterization using transmission electron microscopy (TEM) and scanning electron microscopy (SEM) has been performed. Additionally, assessments of loading capacity, encapsulation efficiency, and drug release profiles of R848 from PLGA-based nanoparticles at physiological pH (7.4) have been carried out [160].

### 3.4. Radiotherapy Enhancements

Radiotherapy is a fundamental treatment modality in cancer therapy, employing ionizing radiation to damage tumor DNA and induce cell death. However, its effectiveness is often limited by the challenge of delivering sufficient radiation doses to tumors while minimizing collateral damage to surrounding healthy tissues [161]. The use of NPs in radiotherapy has emerged as a promising strategy to enhance treatment efficacy by selectively increasing radiation absorption in tumor cells while reducing off-target toxicity. Nanoparticles, particularly those composed of high atomic number (Z) materials like gold and bismuth, serve as radiosensitizers by concentrating radiation energy within tumors, thereby amplifying DNA damage and increasing tumor cell death [161].

Beyond radiosensitization, nanoparticles also offer targeted drug delivery capabilities, allowing for the co-administration of chemotherapeutic agents and radiotherapy enhancers to maximize treatment synergy. By leveraging nanoparticle-based delivery systems, radiotherapy can be fine-tuned to achieve greater tumor specificity, improved therapeutic outcomes, and reduced side effects. This section explores the role of nanoparticles in enhancing radiotherapy, their potential for protecting healthy tissues, and their applications in chemo-radiotherapy and advanced image-guided therapies.

#### 3.4.1. Nanoparticles as Radiosensitizers and Protecting Healthy Tissue

Nanoparticles have been extensively explored as radiosensitizers to enhance the efficacy of radiotherapy by increasing radiation absorption in tumor cells while minimizing damage to surrounding healthy tissues. The principle of radiosensitization is based on the ability of high atomic number (Z) materials, such as gold (Au) and bismuth (Bi), to absorb and scatter ionizing radiation more effectively than biological tissues. This results in localized dose enhancement, amplifying radiation-induced DNA damage within cancer cells and improving treatment outcomes [161]. AuNPs are among the most widely studied radiosensitizers due to their biocompatibility, high X-ray absorption coefficient, and ability to be functionalized for targeted tumor delivery. Their presence within tumor tissues leads to an increased deposition of radiation energy, enhancing double-strand DNA breaks and triggering apoptosis [159,162,163,164]. Moreover, AuNPs can be engineered to selectively accumulate in tumors through surface modifications with targeting ligands, reducing systemic toxicity while increasing therapeutic efficacy [165].

**Figure 6:**
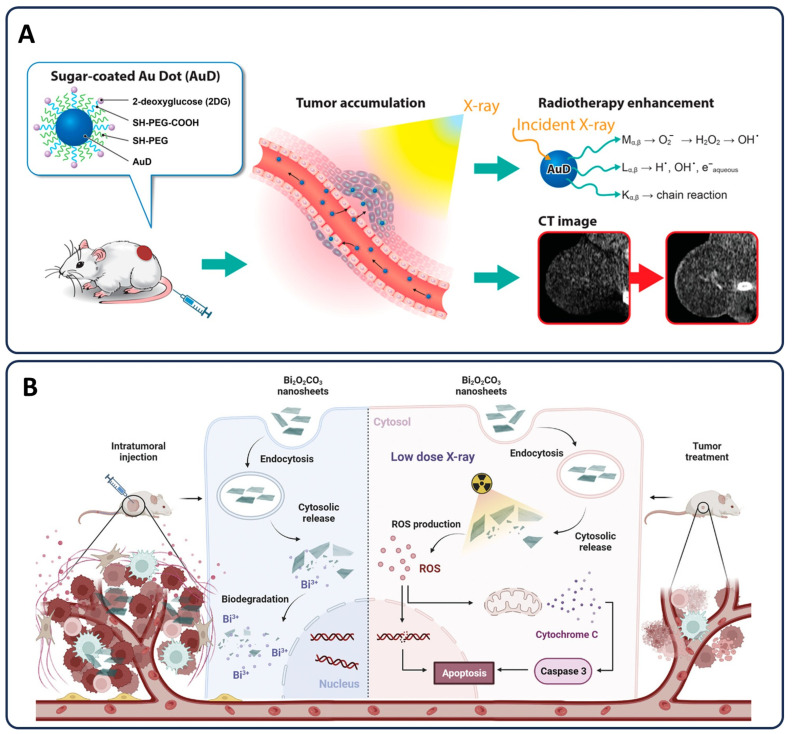
(**A**) Schematic illustrating the synthesis and application of 2-deoxyglucose-coated PEGylated gold nanoparticles (2DG-PEG-AuD). Upon intravenous administration, nanoparticles preferentially accumulate within tumor tissue, enhancing the efficacy of radiotherapy through localized radiosensitization and improved contrast in computed tomography imaging; “Adapted from [168]”. (**B**) Illustration depicting intratumorally administered ultrathin bismuth nanosheets (Bi_2_O_2_CO_3_), their intracellular biodegradation, ROS-mediated apoptotic signaling, and resultant enhancement of tumor radiotherapy under low-dose X-ray irradiation; “Copyright 2025 Wiley. Used with permission from [170]”.

In addition to their role as radiosensitizers, nanoparticles also contribute to the protection of healthy tissues from radiation-induced damage. One approach involves the use of targeted delivery systems that guide nanoparticles specifically to tumor sites, thereby minimizing off-target exposure. Nanoparticles conjugated with tumor-specific peptides or antibodies enhance selective accumulation within cancerous tissues, ensuring that radiation amplification occurs predominantly at the tumor site [166]. Stealth nanoparticles, coated with hydrophilic polymers such as PEG, further reduce systemic toxicity by evading the reticuloendothelial system, prolonging circulation time, and improving tumor targeting via the EPR effect [167]. This dual function of nanoparticles, enhancing radiosensitivity in tumors while reducing radiation exposure to normal tissues, positions them as a powerful tool in the advancement of radiotherapy. Recent advancements in nanoparticle-based radiosensitizers have significantly improved the efficacy of cancer radiotherapy. For instance, 2-deoxyglucose-coated PEGylated gold nanoparticles (2DG-PEG-AuD) accumulate within tumor tissues, enhancing radiosensitization and providing improved contrast in computed tomography imaging (Figure 6A) [168]. PEGylated nanogels encapsulating gold nanoparticles (GNG) have demonstrated increased radiosensitivity and induced apoptosis in various tumor and immortalized cell lines [169]. Ultrathin bismuth nanosheets (Bi_2_O_2_CO_3_) administered intratumorally degrade intracellularly, triggering reactive oxygen species-mediated apoptotic signaling and enhancing tumor radiotherapy under low-dose X-ray irradiation (Figure 6B) [170]. Also, BiO_2−x_ nanosheets augment radiotherapeutic efficacy through interactions involving Auger and Compton electrons, generation of reactive oxygen species, and mitigation of hypoxia-associated radioresistance within tumor cells [171].

#### 3.4.2. Chemo-Radiotherapy: Synergistic Effects and DNA Damage Amplification

The combination of chemotherapy and radiotherapy, known as chemo-radiotherapy, has emerged as an effective strategy to enhance cancer treatment by leveraging the distinct mechanisms of both modalities. While radiotherapy primarily induces DNA damage through ionizing radiation, chemotherapy interferes with DNA replication and repair processes, thereby amplifying radiation-induced cytotoxicity. This synergy enhances tumor cell death, reduces treatment resistance, and improves overall therapeutic outcomes [172]. One of the key mechanisms behind chemo-radiotherapy is DNA damage amplification. Radiotherapy generates double-strand breaks (DSBs) in tumor DNA, which, if left unrepaired, lead to cell death. Chemotherapeutic agents, such as topoisomerase inhibitors (e.g., doxorubicin) and platinum-based drugs (e.g., cisplatin), further exacerbate DNA damage by disrupting DNA repair pathways and increasing radiosensitivity [173,174]. The combination of these effects makes it significantly harder for tumor cells to survive and repair the inflicted damage.

Additionally, nanoparticles have been employed to optimize chemo-radiotherapy by enhancing tumor targeting and minimizing systemic toxicity. By encapsulating both chemotherapeutic drugs and radiosensitizers, nanoparticles enable controlled drug release at the tumor site, ensuring sustained therapeutic effects. For instance, AuNPs loaded with chemotherapeutic agents not only improve radiation absorption due to their high atomic number (Z = 79) but also facilitate localized chemotherapy, further amplifying DNA damage and cell death [175]. Beyond radiosensitization, nanoparticles play a crucial role in DNA repair inhibition, a key approach to improving chemo-radiotherapy effectiveness. Agents such as poly(ADP-ribose) polymerase (PARP) inhibitors, when delivered via nanoparticles, prevent tumor cells from repairing radiation-induced DNA damage, thereby sensitizing them to radiotherapy and increasing treatment efficacy [176,177]. This targeted delivery reduces off-target effects and protects normal tissues from excessive toxicity. Overall, the integration of chemotherapy and radiotherapy, enhanced by nanoparticles, offers a promising approach to cancer treatment by amplifying DNA damage, selectively targeting tumor cells, and overcoming resistance mechanisms. As nanotechnology advances, the potential for more precise and effective chemo-radiotherapy continues to grow.

#### 3.4.3. Advanced Nanoparticle Applications in Radiotherapy

The incorporation of nanoparticles into radiotherapy has changed cancer treatment by improving tumor targeting, enhancing radiosensitivity, and enabling real-time treatment monitoring. Nanoparticles not only act as radiosensitizers, amplifying radiation-induced damage, but also serve as drug carriers that deliver chemotherapy agents in combination with radiation therapy for enhanced therapeutic outcomes [175]. Recent advancements have led to the development of multifunctional nanoparticles that integrate imaging, therapy, and tumor-targeting capabilities to optimize radiotherapy while minimizing side effects on healthy tissues. One of the most widely studied radiosensitizing nanoparticles is AuNPs, which possess a high atomic number and excellent biocompatibility. AuNPs enhance local radiation absorption, leading to an increased dose effect within the tumor while reducing exposure to surrounding normal tissues. Additionally, AuNPs can be functionalized with targeting ligands, such as peptides or antibodies, to ensure selective tumor accumulation and precise delivery of therapeutic agents. Bismuth nanoparticles (BiNPs) represent another class of high-Z materials with dual functionality as radiosensitizers and drug carriers. Bismuth’s strong X-ray absorption enables greater radiation dose deposition at tumor sites, while its high biocompatibility makes it a promising candidate for clinical translation [178]. Furthermore, BiNPs can be loaded with chemotherapeutic agents, providing a synergistic effect between chemotherapy and radiotherapy for improved cancer cell eradication.

Recent breakthroughs in image-guided radiotherapy involve the use of nanoparticle-based contrast agents, which facilitate real-time monitoring of tumor responses to treatment. UCNPs and superparamagnetic iron oxide nanoparticles (SPIONs) have been explored for their potential in multimodal imaging techniques, including MRI, PET, and fluorescence imaging [179]. These nanoparticles enable clinicians to visualize tumor progression and adjust treatment plans accordingly, ensuring precision radiotherapy with minimal collateral damage. Moreover, the emergence of theranostic nanoparticles, which combine therapeutic and diagnostic functions, has opened new possibilities for personalized cancer treatment. These nanoparticles integrate radiosensitizers, chemotherapeutic drugs, and imaging agents into a single nanoplatform, allowing simultaneous tumor visualization, therapy, and monitoring [180]. By tailoring nanoparticles to specific tumor characteristics, clinicians can achieve highly individualized treatment regimens, improving patient outcomes and reducing treatment-related toxicity. As nanotechnology continues to advance, the development of next-generation nanoparticles with enhanced radiosensitizing, imaging, and drug-delivery properties will further refine radiotherapy approaches. These innovations hold great potential in reducing radiation doses, increasing treatment precision, and minimizing adverse effects, making nanoparticle-assisted radiotherapy a cornerstone of future cancer treatment strategies.

### 3.5. Gene Therapy Approaches in Cancer Treatment

Gene therapy represents a transformative approach to cancer treatment by introducing, silencing, or editing genetic material to correct mutations, suppress oncogene expression, or enhance the immune response. Unlike conventional treatments such as chemotherapy and radiotherapy, which directly target rapidly dividing cells, gene therapy intervenes at the molecular level to alter the genetic landscape of cancer cells. The two primary strategies in gene therapy for cancer treatment are gene silencing and gene editing. Gene silencing aims to downregulate oncogene expression using siRNA or short hairpin RNA (shRNA), preventing the translation of proteins that drive tumor progression [181]. On the other hand, gene editing techniques, particularly the CRISPR/Cas9 system, allow for precise modification of DNA sequences, either to correct cancer-associated mutations or to sensitize tumors to existing therapies [182].

Despite the potential of gene therapy, challenges such as off-target effects, inefficient gene delivery, and immune responses have limited its clinical applications. However, advancements in nanoparticle-based delivery systems have significantly improved gene therapy efficacy by enhancing stability, targeting specificity, and reducing systemic toxicity [183]. The integration of nanoparticles in gene therapy has facilitated not only safer and more effective genetic modifications but also novel combination therapies such as chemo-gene therapy, where gene therapy is combined with chemotherapy to overcome drug resistance and improve treatment outcomes [184]. The following subsections explore the mechanisms of gene silencing and editing, the role of chemo-gene therapy in overcoming cancer resistance, and the latest advancements in nanoparticle-mediated gene therapy.

#### 3.5.1. Gene Silencing and Gene Editing in Cancer Therapy

Gene silencing and gene editing are two fundamental approaches in gene therapy for cancer treatment, targeting the genetic basis of tumor progression and drug resistance. These techniques aim to either suppress oncogene expression or correct genetic mutations, thereby enhancing therapeutic efficacy and reducing tumor survival mechanisms.

Gene silencing focuses on inhibiting the expression of cancer-associated genes through siRNA or shRNA. These RNA molecules interfere with messenger RNA (mRNA) translation, effectively preventing the synthesis of oncogenic proteins that drive tumor proliferation and resistance to treatment [182]. siRNA and shRNA specifically bind to target mRNA, leading to its degradation and subsequent inhibition of oncogene translation, thereby sensitizing cancer cells to chemotherapy and other therapies [183]. Targeted oncogenes such as *EGFR*, *Bcl-2*, and *KRAS*, which are frequently overexpressed in various cancers, have been successfully silenced using siRNA-based approaches, demonstrating enhanced tumor suppression and chemosensitization [184]. However, a major challenge in siRNA therapy is its short half-life and poor cellular uptake, necessitating the use of nanoparticle-based delivery systems to improve stability and targeted delivery to tumor cells [185].

**Figure 7:**
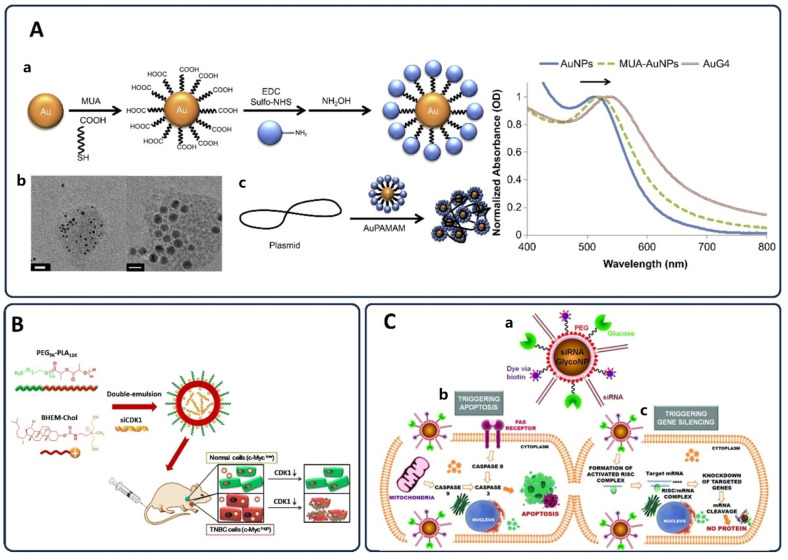
(**A**) (**a**): Diagram illustrating the synthesis of AuPAMAM nanoparticles using the original pH 6 protocol. (**b**): TEM images of generation 4 AuPAMAM (AuG4) nanoparticles complexed with *GFP* plasmid at a 1:30 mass ratio, with DNA visualized using uranyl acetate. Scale bar: 20 nm. (**c**): Schematic showing how AuPAMAM nanoparticles bind and form complexes with plasmid DNA; “Reprinted from [209], Copyright 2015, with permission from Elsevier”. (**B**) A schematic representation of a therapeutic strategy for triple-negative breast cancer (TNBC) characterized by *c-Myc* overexpression, employing *CDK1* siRNA delivery via cationic lipid (BHEM-Chol)-facilitated poly(ethylene glycol)-block-poly(d,l-lactide) (PEG_5_K-PLA_11_K) nanoparticles; “Reprinted from [188], Copyright 2015, with permission from Elsevier”. (**C**) (**a**): Multifunctional siRNA glyconanoparticles (siRNA GlycoNPs) initiate apoptosis in cancer cells. (**b**): They activate cell death pathways by upregulating receptors like Fas and triggering caspases. Fas detects external death signals, activating caspase-8 and downstream effector caspases. Alternatively, mitochondrial stress can activate caspase-9, ultimately leading to caspase-3 activation and programmed cell death. (**c**): Additionally, siRNA GlycoNPs silence genes by engaging the RNA interference pathway, causing mRNA degradation or translation inhibition. “Adapted from [197]”.

The CRISPR/Cas9 gene-editing system has improved cancer therapy by enabling precise modifications in the genome to correct oncogenic mutations or disrupt genes responsible for therapy resistance. CRISPR/Cas9 functions by introducing site-specific double-strand breaks in DNA, which can be repaired through homology-directed repair (HDR) or non-homologous end joining (NHEJ), allowing for the correction or deletion of cancer-driving mutations [186]. This approach has been particularly effective in correcting mutations in tumor suppressor genes such as *TP53* and *BRCA1*, which are frequently mutated in cancers like breast, ovarian, and lung cancer [187]. Moreover, CRISPR/Cas9 has been used to disable drug resistance pathways, making tumors more responsive to chemotherapy and immunotherapy. In the context of triple-negative breast cancer *(TNBC)*, characterized by the overexpression of oncogenes such as c-Myc, targeted gene silencing strategies have been developed. Specifically, cyclin-dependent kinase 1 *(CDK1)* has been identified as a critical regulator of cell cycle progression in TNBC cells. Utilizing cationic lipid-assisted poly(ethylene glycol)-block-poly(d,l-lactide) (PEG_5_K-PLA_11_K) nanoparticles for the delivery of *CDK1*-specific siRNA has shown potential in inhibiting tumor growth by effectively silencing CDK1 expression (Figure 7B) [188].

Despite its potential, challenges such as off-target effects and immune activation remain significant barriers to clinical translation. To address these limitations, nanoparticle-based CRISPR delivery systems have been developed, offering targeted, safer, and more efficient gene editing [189]. Nanoparticles not only protect CRISPR components from degradation but also ensure selective delivery to tumor cells, minimizing unintended genetic modifications in healthy tissues. By integrating siRNA-based gene silencing and CRISPR/Cas9 gene editing with nanoparticle-mediated delivery, researchers have developed highly targeted and effective therapeutic strategies that enhance tumor sensitivity to existing treatments and potentially eradicate resistant cancer cell populations. These advancements pave the way for personalized gene therapies, offering a new dimension in cancer treatment with reduced toxicity and improved patient outcomes.

#### 3.5.2. Chemo-Gene Therapy: Combining Genetic and Chemotherapeutic Approaches

Chemo-gene therapy represents a hybrid strategy that integrates chemotherapy with gene therapy, aiming to enhance therapeutic efficacy while overcoming limitations such as drug resistance and tumor heterogeneity. This approach exploits the cytotoxic effects of chemotherapy alongside the genetic modulation capabilities of gene therapy, leading to improved tumor regression and reduced recurrence rates [187].

The combination of chemotherapy and gene therapy capitalizes on multiple synergistic mechanisms. Gene therapy can downregulate drug-resistance genes, making tumors more susceptible to chemotherapeutic agents. For instance, targeting multidrug resistance protein 1 (*MDR1*), which actively expels chemotherapy drugs from cancer cells, can restore drug sensitivity and enhance the cytotoxic effects of chemotherapy [189]. In addition, gene silencing using siRNA or shRNA can inhibit anti-apoptotic genes such as *Bcl-2*, which promote cell survival, thereby amplifying chemotherapy-induced cell death [190]. Chemotherapeutic drugs, in turn, can induce DNA damage, which is further exacerbated when gene therapy disrupts the tumor’s ability to repair genetic lesions [191].

One of the major challenges in chemotherapy is the emergence of drug-resistant tumor cells that evade treatment. Gene therapy offers a precision approach to reversing resistance mechanisms, enabling chemotherapy to remain effective. By silencing drug-efflux pumps, such as MDR1, or modifying DNA repair pathways via CRISPR/Cas9, tumors can be re-sensitized to standard chemotherapeutics like doxorubicin and cisplatin [192].

Additionally, gene editing technologies have been used to knock out oncogenes that drive tumor growth, enhancing the long-term efficacy of chemotherapy. For example, CRISPR/Cas9 has been applied to disrupt KRAS mutations, which are common in pancreatic and lung cancers, rendering tumors more responsive to treatment [193].

The success of chemo-gene therapy depends on efficient co-delivery of genetic materials and chemotherapeutic drugs, which has been greatly enhanced by nanoparticle-based carriers. Nanoparticles, such as lipid-based carriers, dendrimers, and polymeric nanoparticles, serve as dual-functional delivery platforms that encapsulate both gene-modulating agents (siRNA, CRISPR/Cas9 components) and chemotherapy drugs, ensuring targeted and sustained release within tumor tissues [194].

Lipid nanoparticles (LNPs), widely used in RNA-based vaccines, have been adapted for cancer therapy to simultaneously deliver siRNA and chemotherapeutic agents, enhancing tumor-targeted therapy while minimizing off-target effects [195]. For instance, LNPs have been successfully engineered to co-deliver siRNA targeting MDR1 along with doxorubicin, overcoming drug resistance and significantly improving treatment outcomes [196]. Furthermore, the development of multifunctional siRNA glyconanoparticles (GlycoNPs) has provided a dual approach in cancer therapy. These GlycoNPs not only facilitate targeted gene silencing through the RNA interference pathway but also activate apoptotic pathways via death receptor (Fas)-mediated signaling cascades. This dual mechanism enhances the induction of apoptosis in cancer cells, offering a comprehensive therapeutic strategy [197] (Figure 7C).

Dendrimers, which are highly branched nanocarriers, allow for high loading capacity and precise gene-drug co-delivery, making them promising vehicles for chemo-gene therapy [198]. Polymeric nanoparticles, such as PLGA, offer sustained release profiles, enabling long-term therapeutic effects with reduced toxicity [199]. Among dendrimer-based delivery systems, AuPAMAM nanoparticles have demonstrated efficient plasmid DNA binding and intracellular delivery, as shown in Figure 7A, where gold-dendrimer complexes complexed with GFP plasmids facilitated gene transport through electrostatic interactions and pH-controlled release mechanisms.

Chemo-gene therapy has emerged as a powerful approach to combat aggressive and drug-resistant cancers, with preclinical and early clinical studies demonstrating significant improvements in tumor suppression, patient survival, and reduced side effects. The integration of nanoparticle-mediated delivery systems further enhances the feasibility of translating this approach into clinical practice [200]. As research progresses, customized chemo-gene therapy regimens based on individual tumor genetic profiles will enable personalized cancer treatments, paving the way for highly targeted and effective therapeutic strategies. The continuous evolution of gene-editing tools, such as CRISPR/Cas9, coupled with advancements in nanotechnology, holds immense promise for the future of cancer therapy, offering greater precision, fewer side effects, and long-term disease control.

#### 3.5.3. Nanoparticles for Gene Therapy and Drug Delivery

Nanoparticles have emerged as a transformative platform for gene therapy and drug delivery in cancer treatment by ensuring targeted, efficient, and stable transport of therapeutic agents. Their ability to protect nucleic acids from enzymatic degradation, enhance cellular uptake, and enable precise tumor targeting makes them invaluable in gene-based therapies. Gene therapy, which involves silencing oncogenes, repairing mutations, or enhancing immune responses, faces significant challenges due to the instability and poor cellular uptake of naked nucleic acids. Nanoparticles overcome these limitations by acting as protective carriers that facilitate efficient intracellular delivery while reducing systemic toxicity [196,198].

Several nanoparticle-based systems have been developed to enhance gene therapy approaches. LNPs, widely recognized for their role in mRNA vaccines, have also proven effective for siRNA-mediated gene silencing, such as targeting *MDR1* to restore chemotherapy sensitivity in multidrug-resistant tumors. Dendrimers, highly branched macromolecules with tunable chemical properties, provide a high loading capacity for gene-editing tools like CRISPR/Cas9 while simultaneously delivering chemotherapeutic drugs. Polymeric nanoparticles, including those based on PLGA, PEI, and chitosan, ensure controlled and sustained release of genetic materials, enhancing therapeutic efficacy with minimal side effects. Additionally, inorganic nanoparticles such as gold and magnetic nanoparticles are increasingly functionalized for gene therapy applications, serving as dual-purpose platforms for imaging and therapy while facilitating precise gene delivery [199,200,201,202,203].

The co-delivery of gene therapy components with chemotherapeutic agents through nanoparticles has been a key strategy in overcoming drug resistance and enhancing therapeutic outcomes. For instance, lipid nanoparticles encapsulating both siRNA against MDR1 and doxorubicin have demonstrated significant success in restoring chemotherapy sensitivity in resistant cancer cells. Gold nanoparticles conjugated with CRISPR/Cas9 systems have enabled targeted gene editing within tumor cells, modifying resistance pathways and improving chemotherapy efficacy. Magnetic nanoparticles functionalized with gene-silencing agents offer the advantage of externally guided tumor targeting, ensuring site-specific delivery while minimizing off-target effects [204,205,206,207].

As research advances, nanoparticle-based gene therapy is expected to play a crucial role in personalized cancer treatment. The ongoing development of biocompatible and biodegradable nanocarriers, designed to improve tumor-specific targeting and controlled drug release, holds great promise for clinical applications. Future innovations in nanoparticle formulations will likely lead to more effective, minimally invasive, and patient-specific treatment regimens, further solidifying the role of nanomedicine in revolutionizing cancer therapy [208].

#### 3.5.4. Advances in Targeted Gene Editing and Future Perspectives

The rapid advancements in targeted gene editing have improved cancer therapy, offering precise tools to modify genetic material within tumor cells. The CRISPR/Cas9 system has emerged as one of the most reliable gene-editing techniques, enabling the targeted correction of mutations, deletion of oncogenes, and enhancement of tumor suppressor genes. Unlike traditional cancer therapies, which often have systemic side effects, gene editing provides a highly specific approach to manipulating the tumor genome, potentially leading to long-term remission and reduced recurrence rates [185,186].

One of the primary challenges in gene editing is the efficient and safe delivery of CRISPR/Cas9 components to tumor cells. Naked nucleic acids are prone to rapid degradation and exhibit low cellular uptake. To address these issues, nanoparticles have been extensively explored as carriers for gene-editing tools. LNPs, similar to those used in mRNA vaccines, have demonstrated high efficiency in delivering CRISPR/Cas9 components to cancer cells while minimizing immunogenicity and off-target effects. Polymeric nanoparticles such as PLGA and PEI enhance stability and enable controlled release, improving intracellular gene-editing efficacy. Gold and magnetic nanoparticles have also been functionalized to serve as targeted delivery platforms, allowing for guided gene modification and imaging-assisted precision therapy [187,189,190,191]. The integration of gene editing with other therapeutic modalities is an emerging trend in cancer treatment. Chemo-gene therapy, which combines CRISPR-based modifications with chemotherapeutic drugs, has been particularly effective in overcoming drug resistance. By silencing genes responsible for chemoresistance, such as *MDR1* or *Bcl-2*, gene editing can sensitize tumor cells to chemotherapy, enhancing drug efficacy. Furthermore, modifying genes involved in immune evasion enables the immune system to recognize and attack tumor cells more effectively, paving the way for gene-enhanced immunotherapies [192,193,194,195].

Looking forward, the future of targeted gene editing in cancer therapy is focused on improving precision, minimizing off-target effects, and optimizing delivery systems. The development of advanced CRISPR variants, such as base editing and prime editing, holds great promise for achieving highly specific genetic modifications with minimal unintended consequences. The use of artificial intelligence and bioinformatics in designing gene-editing strategies is expected to enhance target selection and therapy customization. While the clinical translation of gene-editing therapies remains in its early stages, ongoing research in nanoparticle-based delivery systems and genetic engineering is set to redefine cancer treatment. With continued advancements, gene editing could transition from an experimental approach to a mainstream therapeutic modality, offering highly personalized and curative solutions for a wide range of cancers [196].

## 4. Challenges & Limitations

Despite the encouraging potential of nanoparticles in cancer therapy, several challenges and limitations hinder their widespread clinical translation. These challenges span toxicity concerns, scalability issues in manufacturing, regulatory and ethical constraints, and biological barriers affecting therapeutic efficacy [1,210]. Addressing these limitations is crucial to optimizing nanoparticle-based treatments for safe and effective use in oncology [211]. The following sections discuss these obstacles and possible strategies to mitigate them.

### 4.1. Toxicity & Safety Concerns

The long-term safety of nanoparticles remains a major concern in nanomedicine, particularly due to their potential for bioaccumulation and cytotoxicity. Chronic exposure to non-biodegradable nanoparticles, such as AuNPs, has been shown to result in their accumulation in vital organs, particularly the kidneys, due to their resistance to metabolic breakdown and excretion [212]. Studies indicate that nanoparticles around 95 nm in size exhibit the highest renal deposition rate, increasing the risk of kidney stones and fibrosis [212]. Once in circulation, nanoparticles interact with biological macromolecules, including proteins, lipids, and DNA, triggering a cascade of cytotoxic effects such as oxidative stress, inflammation, and apoptosis via molecular pathways like mitogen-activated protein kinases (MAPK) and nuclear factor kappa B (NF-κB) [212,213]. Inhalation of nanoparticles poses additional risks, as studies have linked their pulmonary exposure to oxidative stress, epithelial cell damage, and fibrosis in the respiratory tract [214]. Nanoparticles that penetrate the blood-brain barrier can induce neurotoxicity, including genotoxic effects, neuronal apoptosis, and developmental impairments [214]. Their ability to cross the blood-testis and placental barriers raises additional concerns, as they have been associated with reproductive toxicity, hormonal dysregulation, and germ cell damage [214].

Another major challenge is the potential immunomodulatory effects of nanoparticles, as they can suppress immune responses by altering cytokine and chemokine production, leading to systemic immunosuppression [214]. Long-term studies in animal models have demonstrated the persistence of nanoparticles, particularly in the liver and kidneys, underscoring the necessity of further research to assess their chronic toxicity in humans [215]. Addressing these safety concerns requires rigorous preclinical and clinical evaluations, including the development of biodegradable and targeted nanoparticle formulations that minimize systemic exposure while maximizing therapeutic efficacy.

While nanoparticles have shown great potential in cancer treatment, their toxicity remains a major concern for clinical translation [216]. Toxic effects depend on several factors, including the size, shape, composition, surface charge, and concentration of the nanoparticles [217]. Inorganic nanoparticles, such as AuNPs, silver (AgNPs), QDs, and Fe_3_O_4_, have been shown to induce ROS production, DNA damage, and sometimes organ toxicity in vivo [218,219]. These toxic effects vary widely by material and dose, highlighting the importance of systematic in vitro and in vivo toxicity evaluation [220]. To better compare their safety profiles, Table 2 summarizes recent findings on common inorganic nanoparticles used in cancer therapy.

One effective strategy to reduce nanoparticle toxicity is the use of polymer surface coatings. Common polymers such as PEG, PLGA, and polyvinylpyrrolidone (PVP) have been widely used to increase nanoparticle biocompatibility and circulation time [231,232]. These coatings reduce immune system recognition, decrease inflammatory responses, and help prevent unwanted aggregation [233]. PEG-coated gold nanoparticles show much lower cytokine activation than uncoated particles [234]. Likewise, PVP-coated silver nanoparticles reduce oxidative stress and cytotoxicity in normal tissues [235].

### 4.2. Manufacturing, Quality Control, and Scalability

The transition from laboratory-scale nanoparticle synthesis to large-scale industrial production presents significant challenges due to the stringent requirements for consistency, quality control, and regulatory compliance. Maintaining precise control over nanoparticle size, shape, charge, composition, and physicochemical properties is crucial, as even minor variations can impact bioavailability, stability, and therapeutic efficacy [236]. Nanoparticle manufacturing primarily follows two approaches: the top-down method, which involves breaking down larger structures into nanoscale particles through mechanical or chemical processes, offering better control over particle uniformity [236], and the bottom-up method, which assembles nanoparticles from simpler molecular components but often faces scalability limitations due to batch variability and reaction efficiency [237]. Both methods require extensive optimization to ensure reproducibility and batch-to-batch consistency, especially in medical applications where nanoparticle behavior in biological systems is highly sensitive to surface properties.

To address these challenges, regulatory bodies such as the U.S. Food and Drug Administration (FDA) and the International Council for Harmonisation of Technical Requirements for Pharmaceuticals for Human Use (ICH) have implemented a Quality-by-Design (QbD) framework. This structured approach defines target product attributes, identifies critical physicochemical and biological properties, and incorporates continuous monitoring throughout the manufacturing process to minimize variability and ensure compliance with safety standards [236]. Scalability remains a major hurdle in nanoparticle production. Industrial-scale synthesis must integrate robust quality control measures, including high-throughput characterization techniques such as dynamic light scattering (DLS), TEM, and mass spectrometry, to validate nanoparticle integrity and purity. Additionally, nanoparticle formulations must be optimized for long-term stability, as environmental factors, aggregation, and degradation can affect product shelf life and clinical performance. Addressing these manufacturing and quality control challenges is essential to advancing nanomedicine from experimental research to widespread clinical application.

### 4.3. Regulatory and Ethical Challenges

The rapid development of nanomedicine presents regulatory and ethical challenges that must be addressed to ensure the safe and effective clinical translation of nanoparticle-based therapies. The complexity of nanoparticles, which often exhibit unique physicochemical and biological properties, creates difficulties in classification, standardization, and regulatory approval. Currently, nanomedicine faces regulatory hurdles due to inconsistent safety guidelines, varying analytical methodologies, stability concerns, and discrepancies between in vitro and in vivo results. The FDA and the European Medicines Agency (EMA) have developed regulatory frameworks to assess nanoparticle safety and efficacy, but challenges remain in ensuring reproducibility, systemic biodistribution analysis, and environmental impact assessments [238].

One of the major regulatory concerns is the lack of comprehensive long-term safety data, particularly regarding the systemic accumulation, metabolism, and potential toxicity of nanoparticles over extended periods. Unlike conventional pharmaceuticals, nanoparticles can interact dynamically with biological systems, leading to unforeseen immunological responses, bioaccumulation, or unintended organ deposition. Understanding and mitigating these risks requires rigorous toxicity assessments, standardized testing protocols, and improved preclinical models that accurately predict human responses [236]. Beyond regulatory issues, ethical considerations play a crucial role in nanomedicine development. The integration of nanoparticles in disease detection and treatment raises concerns about privacy, informed consent, equitable access, and potential misuse [239]. For example, advanced nanodiagnostics capable of real-time biomarker monitoring pose questions regarding patient data security and ethical use of predictive health information. Also, disparities in healthcare access may result in unequal distribution of nanotherapeutics, limiting benefits to wealthier populations while leaving underserved communities behind.

Another pressing ethical issue is the use of animal models and human trials in nanoparticle research. Ensuring informed consent, minimizing harm, and developing alternative testing methods are essential to align nanomedicine development with ethical standards. Furthermore, environmental concerns associated with nanoparticle production, disposal, and potential ecological toxicity highlight the need for sustainable manufacturing practices and comprehensive environmental impact assessments [240,241,242]. Addressing these regulatory and ethical challenges requires a collaborative effort between researchers, regulatory agencies, policymakers, and bioethicists to establish standardized safety protocols, improve risk assessment methodologies, and promote equitable access to nanomedicine advancements while safeguarding ethical integrity and patient rights.

### 4.4. Biological Barriers to Nanoparticle Therapy

Despite the remarkable potential of nanoparticles in drug delivery and cancer therapy, their clinical success is often hindered by biological barriers that limit their bioavailability, circulation time, and therapeutic efficacy. These barriers include nonspecific distribution, immune system clearance, tumor microenvironment heterogeneity, and drug efflux mechanisms, all of which vary depending on the disease type, administration route, and patient-specific factors [243]. A major challenge in nanoparticle therapy is premature clearance by the immune system. The mononuclear phagocyte system (MPS), primarily driven by macrophages and monocytes, rapidly recognizes and eliminates nanoparticles based on their size, charge, and surface properties. Nanoparticles that lack stealth coatings or immune-invasive modifications can trigger opsonization, leading to their clearance before reaching the intended tumor site. Certain metallic nanoparticles, such as silver or iron oxide, have been shown to induce ROS production and inflammatory responses, further complicating their therapeutic use [244,245]. Additionally, achieving true stealth behavior remains a major biological barrier to effective nanoparticle delivery. Stealth approaches, typically involving surface modifications like PEGylation, aim to avoid immune clearance and extend circulation time. Nonetheless, despite their widespread use, these systems often face challenges such as variable pharmacokinetics, rapid initial clearance (α-phase), and dose-dependent behavior associated with reticuloendothelial system saturation. Overstating stealth performance, particularly in the absence of early-phase pharmacokinetic assessment, may lead to inaccurate conclusions. Therefore, thorough evaluation of nanoparticle pharmacokinetics across different doses, ideally with real-time monitoring techniques, is critical to overcoming biological barriers and advancing nanoparticle-based therapeutic strategies [246].

Another significant hurdle is tumor microenvironment heterogeneity, which impacts nanoparticle penetration and drug release. Tumors exhibit variability in vascular permeability, extracellular matrix (ECM) density, pH levels, and oxygen availability, all of which influence nanoparticle behavior. For instance, hypoxic regions within solid tumors reduce the efficacy of oxygen-dependent therapies such as PDT, while dense ECM networks can act as physical barriers that hinder nanoparticle diffusion [247]. Studies using murine breast tumor models have demonstrated that nanoparticle transport can be highly inefficient due to these microenvironmental factors, emphasizing the need for engineered nanoparticles that can adapt to tumor heterogeneity [248]. Overcoming these biological barriers requires innovative nanoparticle design strategies. Recent research has explored shape-adaptive nanoparticles that can morph in response to environmental cues, allowing for improved penetration and retention in tumors. Additionally, immune-evasive coatings that mimic host biomolecules, such as cell membrane-coated nanoparticles, have shown promise in reducing immune clearance and extending circulation time [249]. While current research has yet to fully elucidate the direct impact of tumor heterogeneity on nanoparticle performance, integrating bioengineered nanocarriers with real-time adaptability could significantly improve targeted drug delivery and therapeutic outcomes [247]. Addressing these biological barriers is essential for advancing nanoparticle-based precision medicine and ensuring their widespread clinical adoption. Future studies should focus on developing nanoparticles with enhanced tumor-targeting capabilities, immune evasion properties, and microenvironment-responsive drug release to maximize therapeutic efficacy while minimizing off-target effects.

## 5. Future Perspectives

Nanomedicine is on the brink of revolutionizing modern healthcare by addressing long-standing challenges in cancer therapy, drug delivery, and disease monitoring. The future of this field is defined by its shift toward personalized medicine, emerging nanotechnologies, interdisciplinary collaborations, and translational efforts bridging research and clinical applications [250]. Advances in biomarker-driven drug targeting, smart nanoparticles, nanorobots, and artificial intelligence-driven optimization are paving the way for more precise, efficient, and patient-specific treatments. However, these innovations require continuous collaboration between researchers, clinicians, and industry stakeholders to ensure regulatory compliance, scalability, and successful clinical integration. This section explores the key areas shaping the future of nanomedicine and its potential to redefine therapeutic strategies.

### 5.1. Personalized Nanomedicine and Biomarker Integration

Nanomedicine is shifting towards personalized therapy, moving beyond the conventional one-size-fits-all approach. By leveraging a patient’s unique genetic, proteomic, and metabolomic profiles, nanoparticle-based treatments can be customized for maximum therapeutic efficacy and minimal systemic toxicity [251]. Advances in high-throughput sequencing and bioinformatics enable clinicians to tailor nanoparticles to individual variations in disease progression, immune response, and drug metabolism [251]. For instance, ligand-specific lipid nanoparticles can be engineered to encapsulate chemotherapy drugs, targeting specific cancer cells while sparing healthy tissues [252]. The integration of artificial intelligence (AI) and machine learning (ML) further enhances personalized nanomedicine. AI-driven models analyze large datasets to identify biomarker-disease correlations, enabling the optimization of nanoparticle design and treatment regimens [252]. Surface engineering of nanoparticles allows for the precise targeting of diseased cells, reducing off-target effects and improving patient outcomes. Real-time nanosensors embedded within nanoparticles provide continuous physiological feedback, allowing for dynamic treatment adjustments based on real-time biological responses [200].

Biomarker identification plays a crucial role in targeted therapy and disease monitoring. Proteins, nucleic acids, and metabolic markers serve as molecular signatures for detecting disease states and assessing treatment responses [200]. For instance, nanoparticle-based HER2-targeted therapy selectively binds to HER2-overexpressing breast cancer cells, enhancing drug delivery efficacy while minimizing toxicity to normal tissues [253]. In addition, biosensor-functionalized nanoparticles can track biomarker fluctuations, providing clinicians with real-time diagnostic insights and guiding treatment decisions [253,254]. Emerging multiplexed nanoplatforms capable of detecting multiple biomarkers simultaneously are improving precision medicine, enabling early disease detection and therapeutic optimization. The integration of biomarker-responsive nanosystems with advanced nanotechnology is paving the way for more effective, individualized treatment strategies that adapt dynamically to a patient’s evolving disease state [253].

### 5.2. Emerging Nanotechnologies in Medicine

Advancements in nanotechnology are improving medicine, offering next-generation diagnostic and therapeutic solutions that enhance treatment precision and adaptability. Among these breakthroughs, smart nanoparticles and nanorobots stand out as highly responsive, programmable systems designed to improve drug delivery, imaging, and disease management [255]. Smart nanoparticles are engineered to respond to biological and environmental stimuli, including pH, temperature, light, and enzymatic activity [255]. This adaptability allows for controlled drug release, minimizing systemic toxicity while increasing site-specific efficacy. For example, pH-sensitive nanoparticles remain stable under normal physiological conditions but release chemotherapy drugs in the acidic tumor microenvironment, maximizing tumor cell destruction while protecting healthy tissues [256]. Similarly, temperature-responsive nanoparticles are being explored for hyperthermia-based cancer therapy, where externally applied heat triggers localized drug release, amplifying therapeutic effects [256]. In parallel, nanorobots are at the forefront of precision medicine, offering unprecedented accuracy in targeted drug delivery, diagnostics, and microsurgery [257]. These autonomous or externally controlled nanoscale machines navigate through biological environments using chemical, magnetic, or acoustic propulsion systems, allowing them to detect malignant cells and deliver therapeutic payloads exclusively to tumor sites [257]. Some nanorobots are designed with DNA origami-based structures programmed to unfold in response to disease-specific biomarkers, ensuring precise drug deployment while minimizing systemic side effects [258].

Beyond oncology, emerging nanotechnologies are making strides in neurological disorders, cardiovascular diseases, and regenerative medicine. Neuro-nanoparticles engineered to cross the blood-brain barrier are being developed for targeted treatment of Alzheimer’s and Parkinson’s disease [258]. In regenerative medicine, self-assembling nanomaterials promote tissue repair and regeneration, offering promising solutions for wound healing, bone repair, and organ regeneration [259]. As these next-generation nanotechnologies continue to evolve, their potential to redefine medical treatment grows. The integration of AI-driven modeling, bioengineered nanomaterials, and real-time tracking systems will enhance their clinical applications, paving the way for personalized, efficient, and minimally invasive therapies [260].

### 5.3. Interdisciplinary Collaborations in Nanomedicine

The convergence of nanotechnology, oncology, and immunology has revolutionized modern medicine, fostering the development of highly targeted and effective therapeutic strategies. Nanotechnologists contribute by designing smart nanoparticles and nanocarriers for drug delivery, diagnostic imaging, and immune system modulation, while oncologists provide insights into tumor biology, microenvironmental dynamics, and mechanisms of drug resistance [259]. Immunologists further enrich this collaboration by dissecting complex tumor-immune interactions, leading to novel approaches such as nanoparticle-based vaccines, immune checkpoint inhibitors, and CAR-T cell enhancements [260]. These interdisciplinary efforts have paved the way for nanoplatforms with enhanced specificity, allowing for precise tumor targeting while minimizing systemic toxicity. Advances in cancer biosensors and contrast agents now enable real-time disease monitoring, improving early detection and optimizing therapeutic responses [260]. The integration of artificial intelligence (AI) and bioinformatics is enhancing predictive modeling, enabling optimized drug formulations and individualized treatment strategies [261].

Beyond oncology, collaborations between nanomedicine, cardiology, and neurology are leading to innovations in cardiovascular drug delivery, neurodegenerative disease treatment, and regenerative medicine. For instance, nanoparticle-based therapies are being developed to deliver neuroprotective agents across the blood-brain barrier, offering new hope for Alzheimer’s and Parkinson’s disease patients [262]. Similarly, biodegradable nanoscaffolds for tissue engineering and organ regeneration are advancing the field of regenerative medicine [262]. Moving forward, deepening interdisciplinary collaborations will be essential for overcoming current challenges in nanomedicine, including biological barriers, toxicity concerns, and large-scale manufacturing. Expanding partnerships between scientists, engineers, clinicians, and computational experts will accelerate the translation of nanomedical innovations from the laboratory to clinical applications, ensuring safer, more effective, and personalized healthcare solutions [263].

### 5.4. Translational Pathways: Public-Private Partnerships and Clinical Advancements

Bridging the gap between nanotechnology research and clinical applications requires strong public-private partnerships (PPPs) that integrate academia, government agencies, and private industry. These collaborations accelerate the translation of encouraging nanomedical innovations by overcoming challenges in large-scale manufacturing, regulatory approval, and clinical implementation [263]. Academic institutions provide cutting-edge nanoparticle designs and mechanistic insights, while private industry contributes expertise in production, commercialization, and market deployment. Government agencies play a crucial role in funding early-stage research, streamlining regulatory processes, and ensuring safety and efficacy compliance [264]. PPPs also facilitate multi-center clinical trials, allowing for the rapid evaluation of nanomedicines across diverse patient populations. By aligning regulatory frameworks early in development, these partnerships help ensure that nanoparticle-based therapies meet FDA, EMA, and ICH standards efficiently [264]. Moreover, industry involvement helps bridge the “valley of death”, a phase where many technologies fail due to insufficient funding, scalability issues, or lack of regulatory alignment [265].

Recent advancements in nanomedicine commercialization highlight the success of PPPs in advancing personalized and precision medicine. For example, lipid nanoparticle (LNP)- based drug delivery systems, initially developed for RNA therapies, gained rapid approval for COVID-19 vaccines, demonstrating the potential of nanotechnology in accelerated clinical translation [266]. Similarly, collaborations between pharmaceutical companies and research institutions have led to the development of tumor-targeting nanomedicines, improving therapeutic index while reducing systemic toxicity [261]. Looking ahead, PPPs will continue to drive multifunctional nanoparticle platforms for personalized medicine, rare diseases, and global health challenges. By fostering innovation, reducing healthcare costs, and expanding access to advanced nanotechnologies, these partnerships will play a pivotal role in shaping the future of precision medicine and next-generation cancer therapies [264,265].

## 6. Conclusions

The integration of nanotechnology into cancer treatment has improved therapeutic strategies by addressing critical limitations of conventional modalities. Nanoparticles have emerged as powerful tools for enhancing the efficacy of chemotherapy, radiotherapy, immunotherapy, phototherapy, and gene therapy, enabling precise drug delivery, improved tumor targeting, and reduced systemic toxicity. By leveraging their unique physicochemical properties, nanoparticles facilitate synergistic treatment combinations, overcome multidrug resistance, and modulate the tumor microenvironment to enhance therapeutic outcomes. Despite their promise, the clinical translation of nanoparticle-based therapies faces several challenges, including toxicity concerns, immune clearance, large-scale manufacturing complexities, and regulatory hurdles. The long-term biocompatibility and biodistribution of nanoparticles remain key areas of investigation, necessitating rigorous safety evaluations and innovative designs to mitigate potential risks. Additionally, the heterogeneity of tumors presents obstacles to nanoparticle penetration and drug release, underscoring the need for advanced delivery strategies tailored to individual tumor microenvironments.

Future advancements in nanomedicine are expected to focus on personalized cancer therapy, wherein nanoparticle formulations are customized based on a patient’s genetic, proteomic, and metabolic profiles. The integration of artificial intelligence and machine learning will further enhance precision medicine by optimizing nanoparticle formulations and predicting treatment responses. Emerging technologies such as smart nanoparticles, nanorobots, and gene-editing nanoplatforms hold great potential for expanding the scope of nanomedicine beyond oncology, into neurology, cardiology, and regenerative medicine. Interdisciplinary collaborations among nanotechnologists, oncologists, immunologists, and bioengineers will be crucial in advancing nanoparticle-based therapies. Public-private partnerships will play a pivotal role in accelerating the transition from laboratory research to clinical applications, addressing scalability challenges, and ensuring regulatory compliance. As nanomedicine continues to evolve, the focus must remain on developing safe, effective, and accessible nanoparticle therapies that improve patient outcomes while minimizing adverse effects. By overcoming current limitations and embracing emerging innovations, nanotechnology has the potential to redefine cancer treatment paradigms, offering highly targeted, minimally invasive, and more effective therapeutic solutions. Continued research and collaboration will be instrumental in realizing the full potential of nanoparticles in oncology, paving the way for the next generation of precision cancer treatments.

## Figures and Tables

**Table 2:** Toxicity of Commonly Used Inorganic Nanoparticles in Cancer Therapy.

Nanoparticle Type	Toxicity Effects	Affected Systems	Notes on Mitigation	Ref.
AuNPs	ROS generation, DNA damage, pro-inflammatory effects	Liver, kidney, and blood cells	PEGylation reduces immune clearance and inflammation	[221,222]
AgNPs	High oxidative stress, mitochondrial damage, genotoxicity	Liver, lungs, immune cells	PVP coating reduces cytotoxicity	[223,224]
Fe_3_O_4_	Iron overload, oxidative stress, ROS	Brain, liver, macrophages	Dextran or PEG coating enhances biocompatibility	[225,226]
QDs	Heavy metal ion release (e.g., Cd^2+^), long-term toxicity	Liver, spleen, reproductive organs	Silica/polymer coating prevents leaching	[227,228]
Zinc Oxide (ZnO NPs)	High ROS production, inflammatory response	Lung, liver, skin	Smaller size + surface modification lowers toxicity	[229,230]

## List of Abbreviations

APCs	Antigen-Presenting Cells
AuNPs	Gold Nanoparticles
BiNPs	Bismuth Nanoparticles
CAR-T	Chimeric Antigen Receptor T Cells
CRISPR/Cas9	Clustered Regularly Interspaced Short Palindromic Repeats/CRISPR-associated protein 9
CTLs	Cytotoxic T Lymphocytes
CTLA-4	Cytotoxic T-lymphocyte–associated Antigen 4
DAMPs	Damage-Associated Molecular Patterns
DCs	Dendritic Cells
DLS	Dynamic Light Scattering
EGFR	Epidermal Growth Factor Receptor
EMA	European Medicines Agency
EPR	Enhanced Permeability and Retention
FDA	U.S. Food and Drug Administration
Fe_3_O_4_	Iron(II,III) Oxide
GM-CSF	Granulocyte-Macrophage Colony-Stimulating Factor
HMGB1	High-Mobility Group Box 1
ICD	Immunogenic Cell Death
ICIs	Immune Checkpoint Inhibitors
IFN-γ	Interferon Gamma
IL-2	Interleukin-2
LNPs	Lipid Nanoparticles
LPHNPs	Lipid-Polymer Hybrid Nanoparticles
MAPK	Mitogen-Activated Protein Kinase
MDR	Multidrug Resistance
MDSCs	Myeloid-Derived Suppressor Cells
MOFs	Metal-Organic Frameworks
MRI	Magnetic Resonance Imaging
NF-κB	Nuclear Factor kappa B
NHEJ	Non-Homologous End Joining
NP(s)	Nanoparticle(s)
PEG	Polyethylene Glycol
PEI	Polyethylenimine
PET	Positron Emission Tomography
PLGA	Poly(lactic-co-glycolic acid)
PDT	Photodynamic Therapy
PD-1	Programmed Cell Death Protein 1
PD-L1	Programmed Death-Ligand 1
PTT	Photothermal Therapy
PVP	Polyvinylpyrrolidone
QbD	Quality-by-Design
QDs	Quantum Dots
ROS	Reactive Oxygen Species
shRNA	Short Hairpin RNA
siRNA	Small Interfering RNA
SPR	Surface Plasmon Resonance
SPIONs	Superparamagnetic Iron Oxide Nanoparticles
TAAs	Tumor-Associated Antigens
TEM	Transmission Electron Microscopy
TLR	Toll-Like Receptor
TME	Tumor Microenvironment
UCNPs	Up-Conversion Nanoparticles
VEGF	Vascular Endothelial Growth Factor
